# An Interactive Workshop on Managing Dysphagia in Older Adults With Dementia

**DOI:** 10.15766/mep_2374-8265.11223

**Published:** 2022-03-02

**Authors:** Nicole Mushero, Lindsay B. Demers, Ryan Chippendale

**Affiliations:** 1 Assistant Professor, Section of Geriatrics, Department of Medicine, Boston University School of Medicine; Physician, Department of Geriatrics, Boston Medical Center; 2 Assistant Professor, Section of Geriatrics, Department of Medicine, Boston University School of Medicine

**Keywords:** Dementia, Dysphagia, Evidence-Based Medicine, Case-Based Learning

## Abstract

**Introduction:**

Nearly six million American adults live with dementia, and dysphagia is a common comorbidity impacting their nutrition and quality of life. There is a shortfall in the number of geriatricians available to care for older adults. Thus, primary care physicians should be equipped with the knowledge to adequately care for the geriatric population. Modified diets are routinely prescribed for patients with dementia despite limited evidence that they protect patients from the sequelae of dysphagia and some suggestion of poor side-effect profiles.

**Methods:**

We created a onetime, interactive, case-based session to educate medical residents on how to evaluate and treat dementia-associated dysphagia and address the discrepancy between the limited evidence for dietary modifications and their routine use. The session had a mixture of small-group discussion and didactic learning as well as a participatory component during which learners were able to sample thickened liquids.

**Results:**

The session was implemented in an established primary care curriculum. Based on survey responses, which were obtained from 15 out of 17 participants, the session significantly improved participants’ knowledge of dysphagia-associated dementia and increased their comfort with caring for patients with dysphagia.

**Discussion:**

Dementia-associated dysphagia, although an increasingly common clinical problem, remains an underexamined area of medicine. We successfully implemented a session on this topic for internal medicine residents on the primary care track. Limitations included generalizability due to the small number of residents in the course and inability to gather sufficient data to see if knowledge learned was sustained over time.

## Educational Objectives

By the end of this workshop, learners will be able to:

1. Appropriately evaluate patients with dementia for dysphagia.
2. Describe the available evidence regarding dietary modifications for dysphagia.
3. Evaluate the risks associated with thickened liquids.
4. Facilitate conversations with patients and families around the risks and benefits of dietary modifications and alternatives.

## Introduction

Dementia is a common phenomenon in older adults, with nearly six million older adults living with the disease in the United States, a number that is expected to double in the next 20 years.^1^ Alzheimer's dementia (AD), the most common form of dementia, accounts for two-thirds of cases.^2^ Nearly all patients with AD deal with some degree of dysphagia, as it tends to arise early in the disease process. Dysphagia impacts the ability of a patient to obtain adequate nutrition and affects the patient's quality of life.^3^ Additionally, dysphagia has been associated with increased morbidity and mortality. As many as 15% of all older adults are estimated to be affected by dysphagia, with that number rising to 45% among people with dementia who are in long-term care settings.^4^ In one prospective study in which patients with AD were screened for dysphagia with videofluoroscopy, only four out of 25 had normal swallow function, and the degree of swallow abnormality correlated with the severity of dementia.^5^

Understanding and teaching around the prevalence and impact of dysphagia in our older adult population are important because of risks to the patient, such as malnutrition and pneumonia, and also because the number of dedicated providers trained in geriatrics will be insufficient to provide the care patients with dementia need.^4,6^ As has been widely cited, many older adults will be taken care of by primary care doctors who have limited training in geriatric syndromes. Although essential geriatric competencies for internal and family medicine residents that touch on many key aspects of geriatric care (e.g., delirium, polypharmacy, and advance care planning) have been established, it is unclear how formally the components are incorporated into general primary care curricula.^7,8^ Additionally, not all common aspects of care for older adults, such as dysphagia, are encapsulated in these competencies.

Currently, patients with dementia who are found to have dysphagia are commonly prescribed dietary modifications such as thickened liquids (with consistencies described as nectar or honey thick) or texture-modified foods (e.g., mechanical soft, puree, ground). However, increasing evidence suggests that these dietary modifications do not lead to decreases in harmful sequelae of aspiration, such as pneumonia, yet may have potential adverse side effects.^9^ Initial evidence for the efficacy of thickened liquids in preventing aspiration came from videofluoroscopic studies that showed a reduction of aspiration events with nectar and pudding boluses, findings that have been replicated with other populations in further studies.^10,11^ However, it was unclear what the clinical correlation was until a 2008 study by Robbins and colleagues that compared ingestion of thickened liquids (nectar and honey thick) with ingestion of thin liquid with postural intervention (chin tuck). The study looked at incidence of pneumonia over 3 months and found no difference in incidence for thickened liquids versus chin-tuck groups.^12^ Further studies supported that those with access to free water had no more risk of developing pneumonia than those without.^9,13,14^ However, the modified diets showed evidence of potential harm in the form of increased risk of urinary tract infection and dehydration, and studies that have looked at quality-of-life scores have shown significant reduction for those on thickened liquid diets.^13^ Additionally, there are no data looking at the effect of texture modification of food (e.g., mechanical soft, puree) on incidence of aspiration pneumonia or other sequalae of aspiration. Despite this, dietary modifications are routinely prescribed for older adults with dementia and dysphagia.

We designed an interactive, case-based, educational workshop for primary care internal medicine residents describing how to evaluate dysphagia in older adults with dementia, which also detailed a review of literature around dietary modifications since these are commonly prescribed to older adults despite limited data describing benefit. We aimed to increase confidence around stopping the use of these interventions and having discussions with patients and families around the risks, benefits, and alternatives such as postural modifications and coached eating.^9,12^ We found no similar interventions described in the literature. An extensive search found educational material primarily related to oral health, as well as one publication targeted at medical student history taking around dysphagia, as opposed to diagnosis and management.^15,16^ We specifically designed a case-based session in order to promote interactivity, prepare residents for what an actual clinical scenario might be like, and give them the opportunity to practice the clinical decision-making and communication skills necessary to counsel families.^17^

## Methods

We developed the session as a result of our experience as geriatricians frequently encountering older adults with dementia who had been prescribed modified diets. These modified diets seemed burdensome and unappealing, and a literature review found that their routine use was not supported by evidence. Given the lack of evidence supporting modified diets combined with their common presence in everyday practice, an educational intervention seemed appropriate.

The target audience for this workshop was internal medicine or family medicine residents, as well as advanced practice providers such as physician assistants or nurse practitioner trainees planning to pursue a career primarily focused in primary care. The curriculum could also be modified for those pursuing hospital medicine, neurology, or other subspecialties that might encounter patients with dementia and dysphagia with regular frequency. The only prerequisite was a basic knowledge of dementia and oropharyngeal dysphagia. The ideal site for implementation was within an already established didactic curriculum allowing for small-group breakouts.

### Logistics

Our session had 17 participants, which was an ideal size for active participation; however, the session size could be slightly smaller or larger (10–25). The residents in our group were a mixed PGY cohort (approximately equal numbers of PGY 1, 2, and 3). Teaching staff included one geriatric medicine fellow and one attending geriatrician. The session was incorporated into an established curriculum for the residents. Our residents were members of the internal medicine primary care track, but, as mentioned above, learners could be from a variety of specialties managing similar patients. Subsequently, this session has been modified to be appropriate for the fourth-year medical student level.

This was a onetime session lasting 90 minutes to give time for participants to fill out the pre- and postsurvey (Appendix A) during the session to maximize completion rates. Removing the survey from the session would make the entirety of the workshop 75 minutes. We arranged the learners in four small groups (four to five people each) around tables in such a way that they could also see the slide presentation (Appendix B). A computer and projector were needed to present the didactic portions of the workshop.

We started the session with a patient case and question to facilitate discussion. While working on this patient case, residents completed a thickened liquid participatory component (thickened liquid powder, juice, cups, spoons) to help inform their decisions. Residents then completed a second patient case. Questions throughout the session focused on diagnosis and management plans that the residents were to create based on the patient case. Between small-group breakouts and large-group debriefs, facilitators gave mini-lecture didactics on the core concepts of dysphagia in older adults. The facilitator's guide (Appendix C) provided a detailed breakdown of the session.

### Evaluation

Using a pre-/postsurvey, we compared residents’ knowledge and attitudes around dysphagia in older adults with dementia before and after the interactive, educational session. We attempted to obtain information about whether residents retained knowledge from the session by administering a delayed postsurvey several months after the session. However, the response rate was low (seven out of 17), so those data have not been included.

Two types of questions were administered to the residents to measure pre-/postworkshop changes in the following domains: (1) attitudes regarding the importance of managing patients with dysphagia in current and future practice (with higher numbers indicating increased comfort) and (2) knowledge (with a higher number indicating correctness). The three attitudinal items had responses on two 5-point scales. One scale, which measured comfort with managing patients with dysphagia, ranged from *I do not feel comfortable managing patients with dysphagia* (1) to *I feel comfortable managing patients with dysphagia independently* (5). The other scale measured the importance of managing patients with dysphagia in current and/or future practice and ranged from *not important at all* (1) to *very important* (5). The five knowledge statements were answered on a scale of *strongly agree* (1) to *strongly disagree* (7) for ease of measuring changes in response. The survey also included nine knowledge questions with correct or incorrect answers, which we developed independently.

In order to compare attitudinal and knowledge changes from pre- to postsurvey, we used a series of nonparametric sign tests appropriate for data obtained from Likert-type responses, with significance at *p* < .05. The one knowledge statement “Thickened liquids can cause adverse events” was reverse coded. We also used a paired-samples *t* test to analyze data arising from answers with correct or incorrect answer choices.

Residents also filled out anonymous evaluations assessing the quality of the session. We reviewed these informally without collating the data, and so, we have not included these data in our results.

## Results

Of participating residents, 15 out of 17 completed the pre- and postsurveys. As shown in the Table, participants who completed the surveys (*n* = 15) reported a significant increase in comfort with managing patients with dementia with dysphagia (*M* = 2.3, *SD* = 0.6, to *M* = 3.5, *SD* = 0.5) and a significant increase in understanding of the importance of caring for patients with dysphagia in their current practice (*M* = 4.3, *SD* = 0.7, to *M* = 4.7, *SD* = 0.5). There was no significant change in residents' attitudes around whether managing dysphagia was important for their future practice. With regard to knowledge change, we saw significant improvements in mean responses for four out of the five statements (Table). Most responses to the statement regarding patients being on a modified diet did not change from pre- to postsurvey (10 ties).

**Table.**
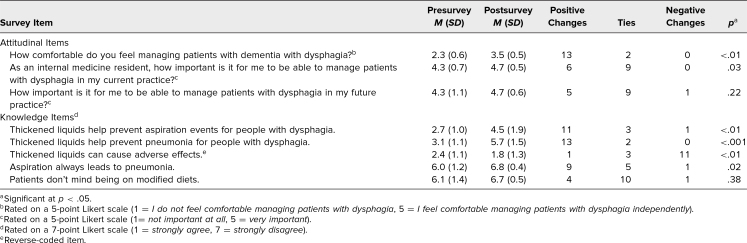
Results From Pre- and Postsurvey (*N* = 15)

The remainder of knowledge items (items 1–9 in Appendix A) were scored as correct/incorrect and converted into the percentage correct at each time point. Participants’ overall knowledge scores increased significantly from a baseline mean of 0.7 (*SD* = 0.1) to a mean of 0.9 (*SD* = 0.1), *t*(14) = 5.89, *p* < .001.

## Discussion

Dysphagia is a common problem in patients with dementia, a population that is only increasing. Geriatricians alone will not be able to provide care for the number of older adults experiencing dementia-associated dysphagia, and thus, education of future primary care providers on common geriatric syndromes is crucial. We created a brief, singular education module to educate internal medicine residents on the evaluation and treatment of dysphagia in older adults. The session was interactive and case-based, teaching methods that are valued by adult learners. Residents rated this session highly, as demonstrated through the anonymous reviews provided by the program director. We saw significant increases in knowledge and attitudes of those participating in the educational module. Although we had hoped to assess whether knowledge gains would be sustained over time, the response rate to a delayed postintervention survey was insufficient for analysis and inclusion here. The insufficient response also limited our ability to assess whether learners had developed comfort with having conversations with patients and families around continuing or ceasing dietary interventions, which was one of our educational objectives.

One change we measured was a shift in learner attitudes around the importance of understanding and managing dysphagia for the residents’ current and future practices. We saw a shift in learner comfort for current practice but not for future practice. One reason for this could have been that although the residents in our audience were in the primary care track, only 41% (seven out of 17) answered that they were planning to go into primary care or some facet of general internal medicine. The other members of the group answered that they were planning to pursue a primary care subspecialty (*n* = 7), a nonprimary care subspecialty (*n* = 2), or were undecided (*n* = 1). As such, a significant portion of the group may have felt that content around dementia and dysphagia was not as relevant to their future practice. It was also possible that we did not accurately convey the scope of the problem and likelihood of encountering dementia-associated dysphagia in most areas of medicine, including subspecialties.

The other aspect of change that we measured was in knowledge base. We saw a statistically significant increase in correct answers on knowledge-based questions from pre- to postsurvey. In particular, residents were able to understand the nuanced difference that the use of thickened liquids does help prevent aspiration on videofluoroscopy but does not appear to prevent sequelae such as pneumonia (Table). The only knowledge question that did not increase was about patient experience with thickened liquids. This may be because residents already perceived that patients do not enjoy modified diets.

The biggest limitation for this session was in the small sample size. Because this session was done with only one group of residents in one program, it is hard to know if other sessions would be similarly successful in addressing the knowledge gap that this session sought to address. Ultimately, we hope to repeat this session to better understand its effect. This will also allow better understanding of whether all of the educational objectives for this session were accomplished.

Overall, this education module is easy to implement and addresses a knowledge gap concerning a common clinical syndrome that residents are likely to encounter in their practices, whether they decide to stay in primary care or go into a subspecialty. We saw a significant change in knowledge and attitudes around the importance of dementia-associated dysphagia and methods used to diagnose and treat this condition, although the generalizability of these findings is limited by the small sample size. There are very few limitations to incorporating this session into a primary care curriculum, as it is a relatively short, singular session that can be taught and facilitated by one to two faculty members. No costly supplies are needed, as the materials for the experiential thickened liquid component are inexpensive and can be eliminated if there is no funding.

Future directions for this curriculum include broadening the audience to include nonprimary care track internal medicine and family medicine residents as well as potentially piloting the workshop with interprofessional trainees. With a larger audience, we hope to better measure the magnitude of learning and its sustainability over time.

## Appendices


Pre- and Postsurvey.docxDysphagia in Dementia.pptxFacilitator Guide.docx
*All appendices are peer reviewed as integral parts of the Original Publication.*
