# Supplementary material for: An Interactive Workshop on Managing Dysphagia in Older Adults With Dementia
Source: MedEdPORTAL. 2022 Mar 2;18:11223. doi: 10.15766/mep_2374-8265.11223 (PMC8888763; doi:10.15766/mep_2374-8265.11223)
Supplement: Supplementary file 1 — Pre- and Postsurvey.docxDysphagia in Dementia.pptxFacilitator Guide.docx [file mep_2374-8265.11223-s001.zip › B. Dysphagia in Dementia.pptx]

## Slide 1
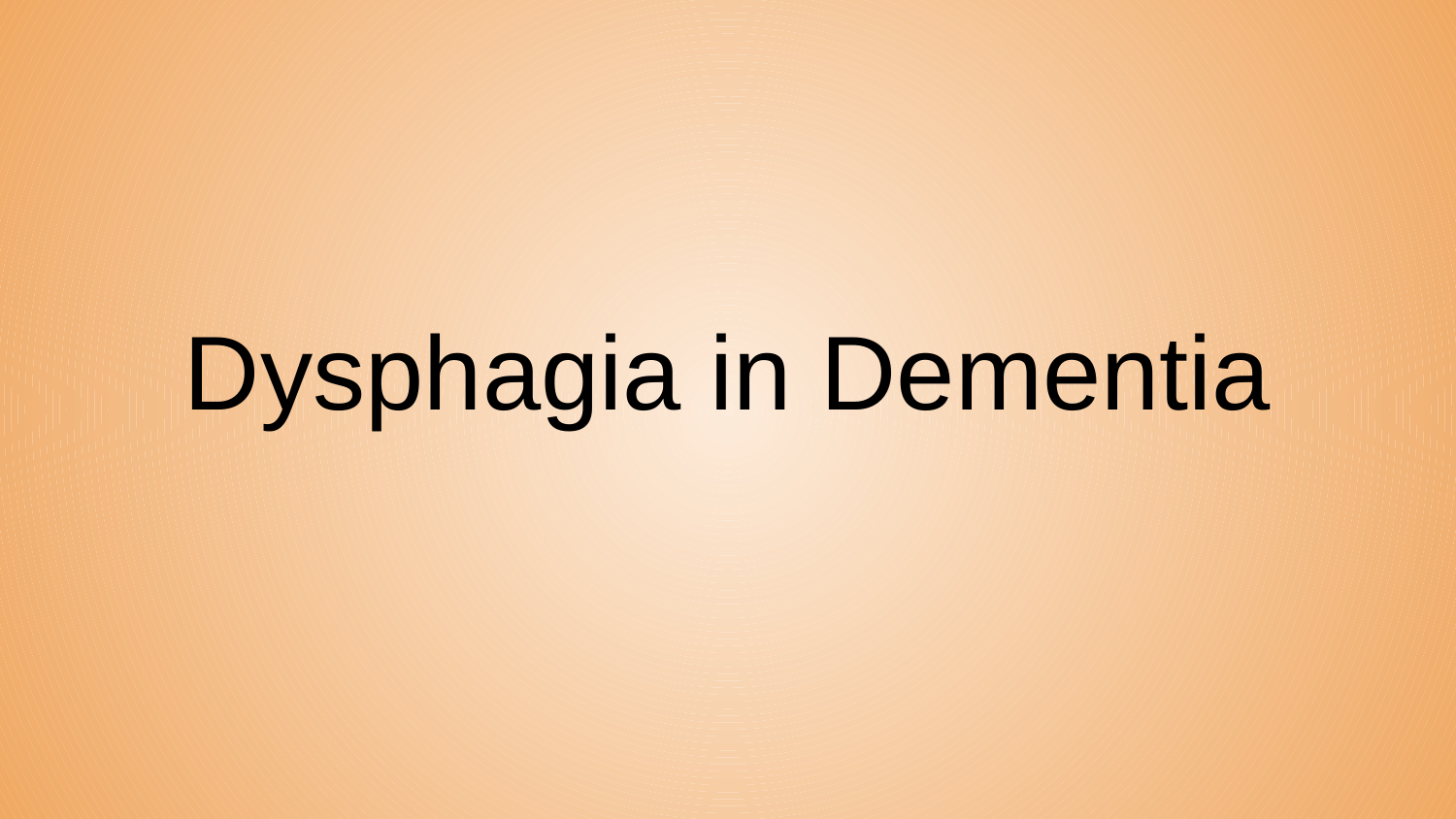

# Dysphagia in Dementia

## Slide 2
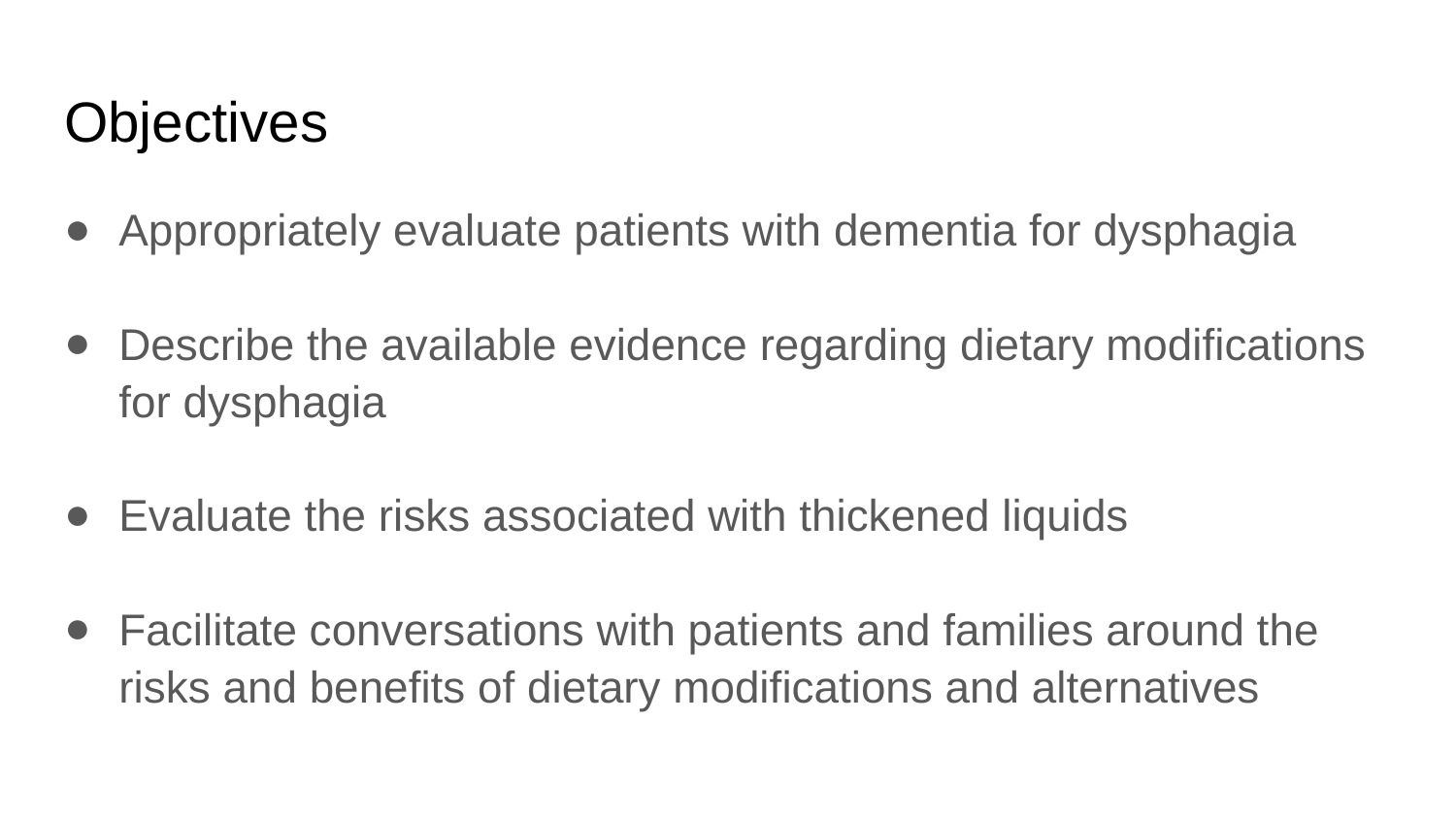

# Objectives
Appropriately evaluate patients with dementia for dysphagia
Describe the available evidence regarding dietary modifications for dysphagia
Evaluate the risks associated with thickened liquids
Facilitate conversations with patients and families around the risks and benefits of dietary modifications and alternatives

## Slide 3
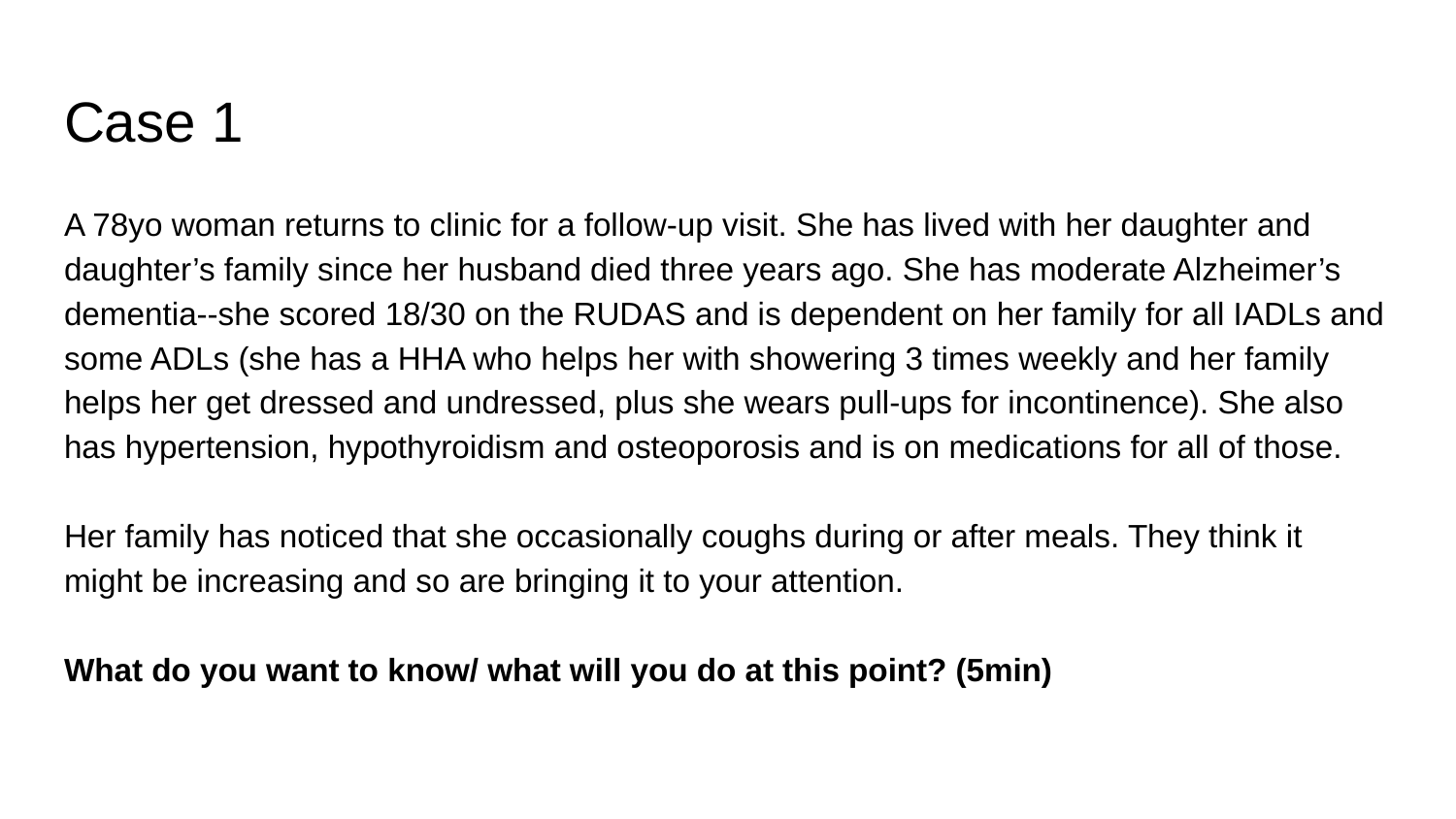

# Case 1
A 78yo woman returns to clinic for a follow-up visit. She has lived with her daughter and daughter’s family since her husband died three years ago. She has moderate Alzheimer’s dementia--she scored 18/30 on the RUDAS and is dependent on her family for all IADLs and some ADLs (she has a HHA who helps her with showering 3 times weekly and her family helps her get dressed and undressed, plus she wears pull-ups for incontinence). She also has hypertension, hypothyroidism and osteoporosis and is on medications for all of those.
Her family has noticed that she occasionally coughs during or after meals. They think it might be increasing and so are bringing it to your attention.
What do you want to know/ what will you do at this point? (5min)

## Slide 4
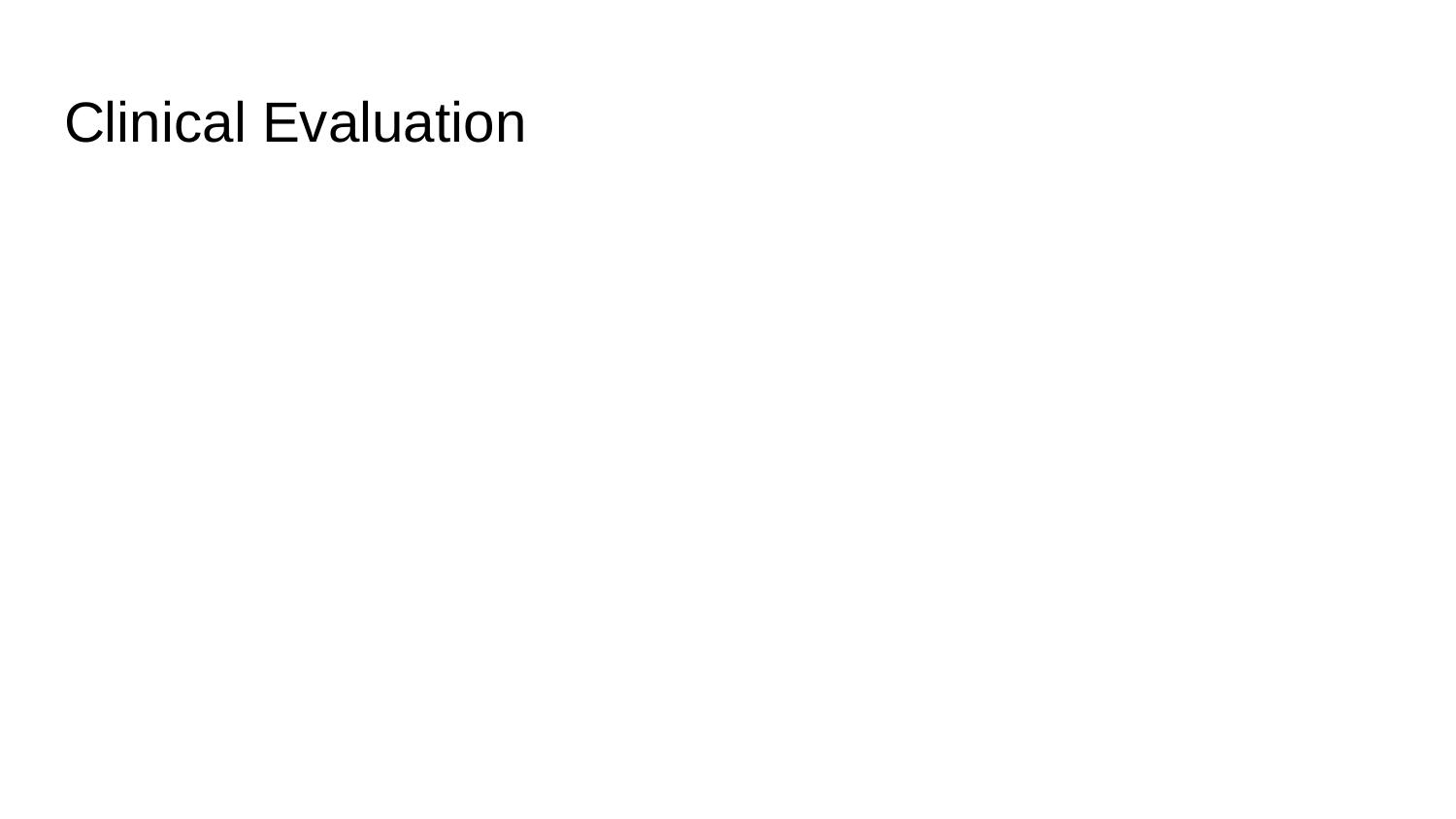

# Clinical Evaluation

## Slide 5
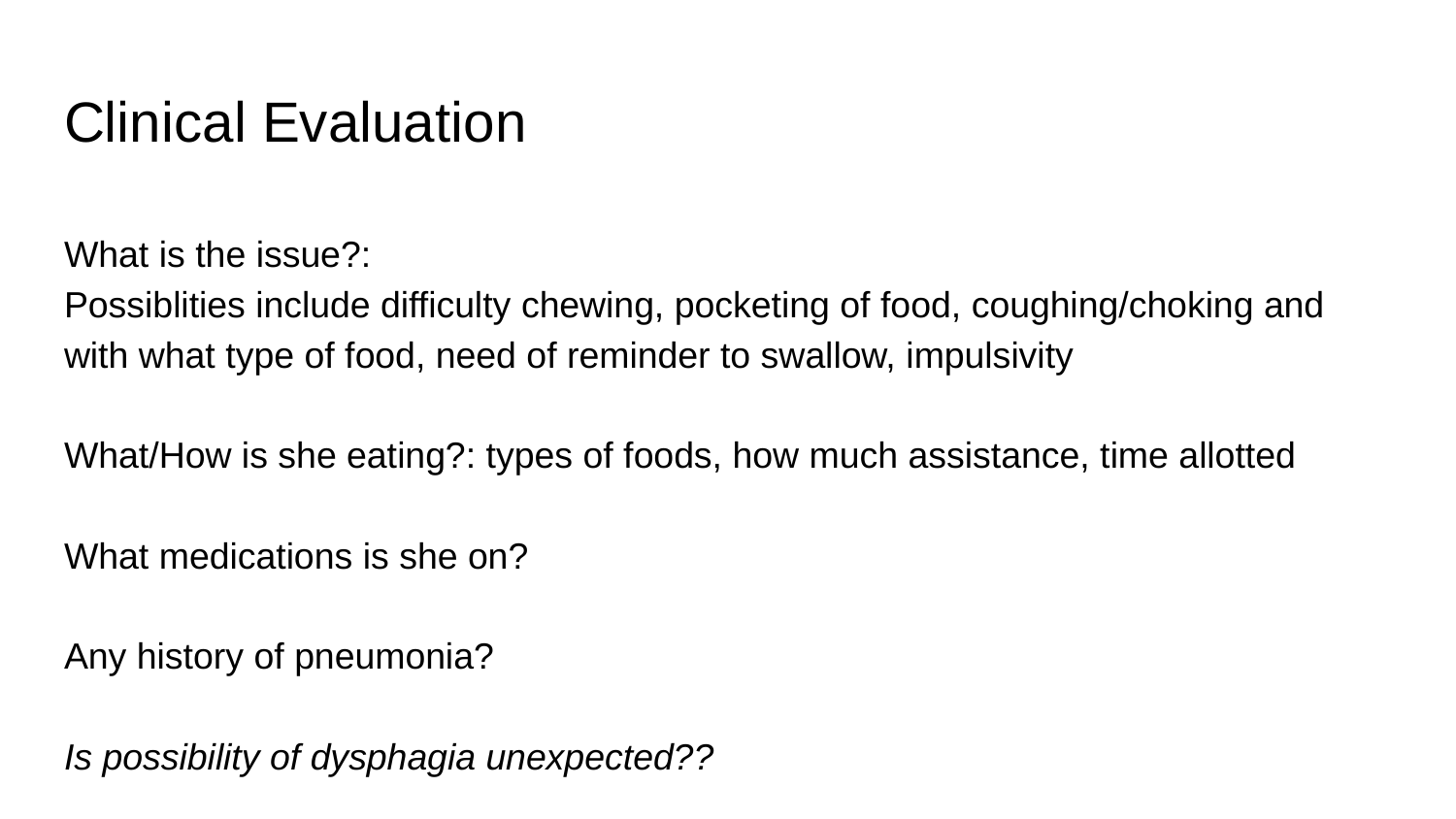

# Clinical Evaluation
What is the issue?:
Possiblities include difficulty chewing, pocketing of food, coughing/choking and with what type of food, need of reminder to swallow, impulsivity
What/How is she eating?: types of foods, how much assistance, time allotted
What medications is she on?
Any history of pneumonia?
Is possibility of dysphagia unexpected??

## Slide 6
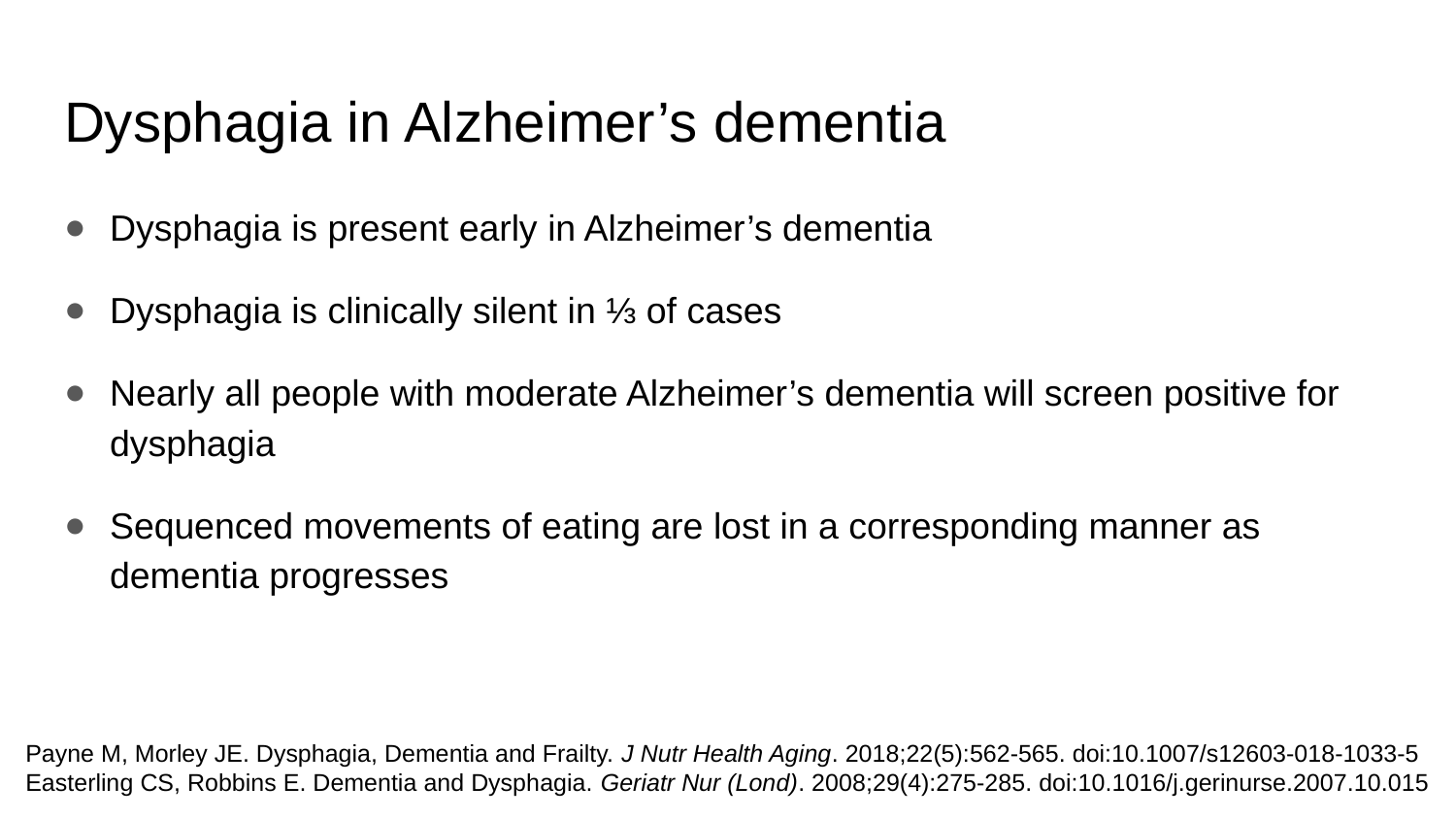

# Dysphagia in Alzheimer’s dementia
Dysphagia is present early in Alzheimer’s dementia
Dysphagia is clinically silent in ⅓ of cases
Nearly all people with moderate Alzheimer’s dementia will screen positive for dysphagia
Sequenced movements of eating are lost in a corresponding manner as dementia progresses
Payne M, Morley JE. Dysphagia, Dementia and Frailty. J Nutr Health Aging. 2018;22(5):562-565. doi:10.1007/s12603-018-1033-5
Easterling CS, Robbins E. Dementia and Dysphagia. Geriatr Nur (Lond). 2008;29(4):275-285. doi:10.1016/j.gerinurse.2007.10.015

## Slide 7
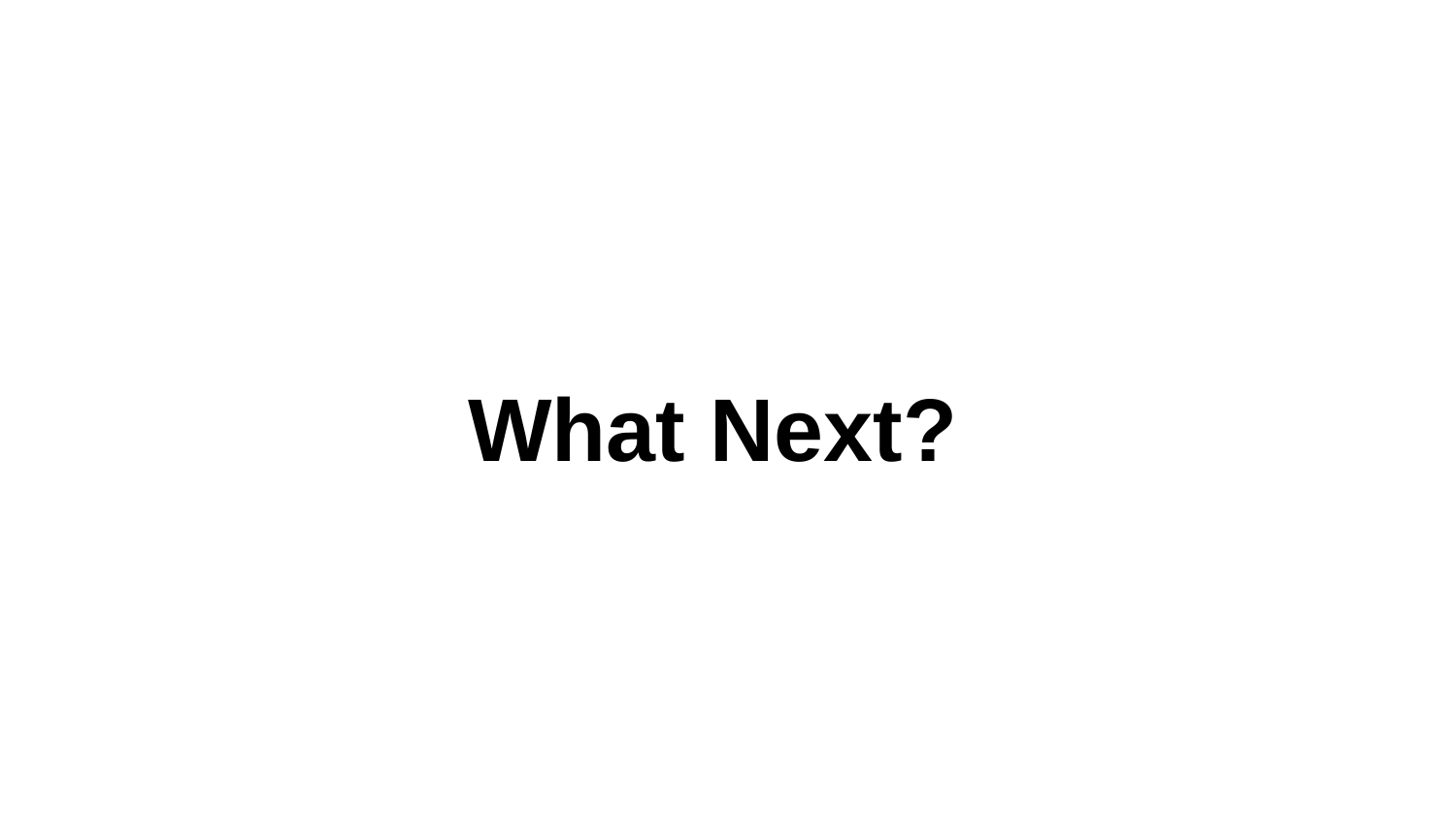

#
 		 What Next?

## Slide 8
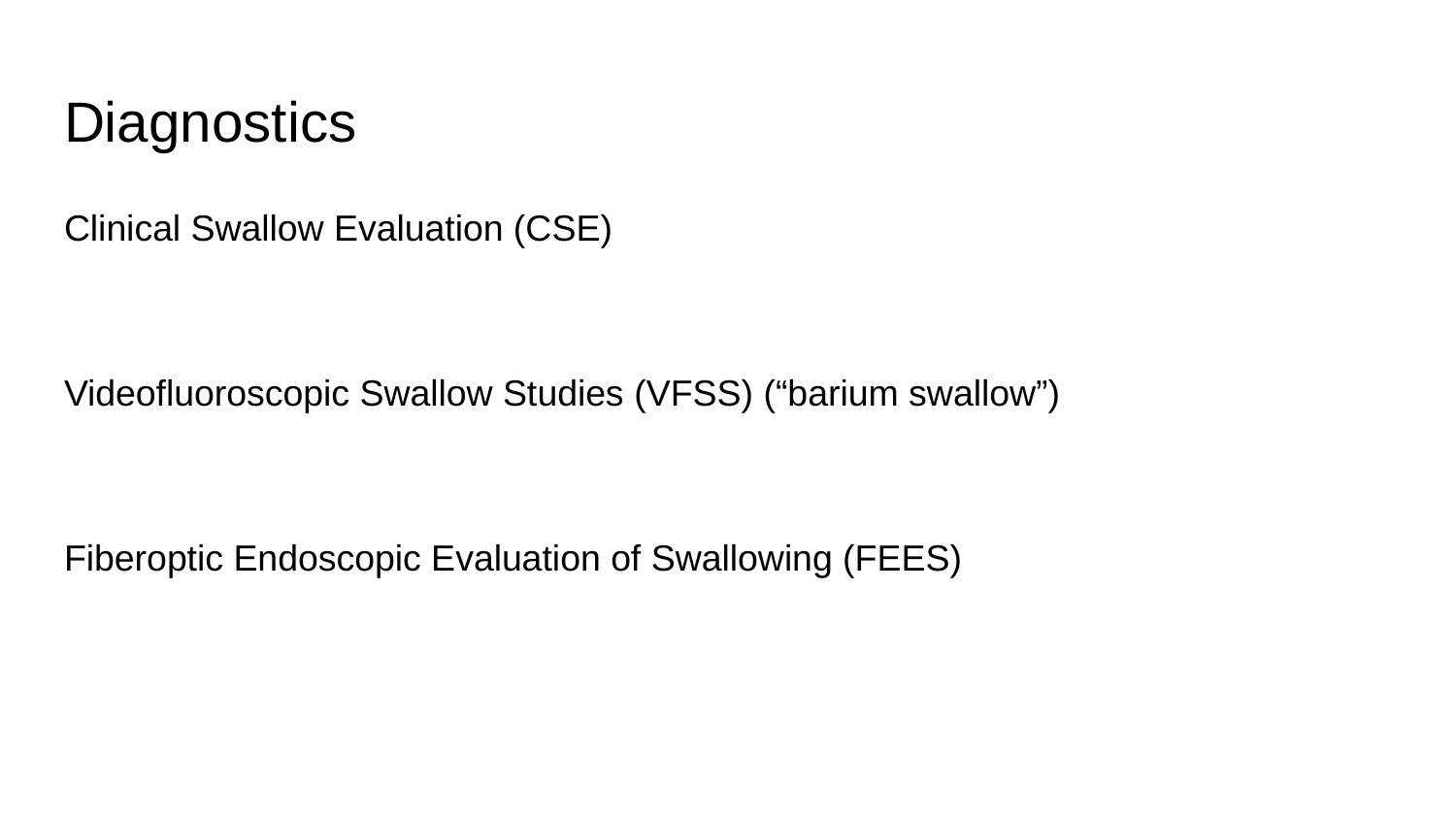

# Diagnostics
Clinical Swallow Evaluation (CSE)
Videofluoroscopic Swallow Studies (VFSS) (“barium swallow”)
Fiberoptic Endoscopic Evaluation of Swallowing (FEES)

## Slide 9
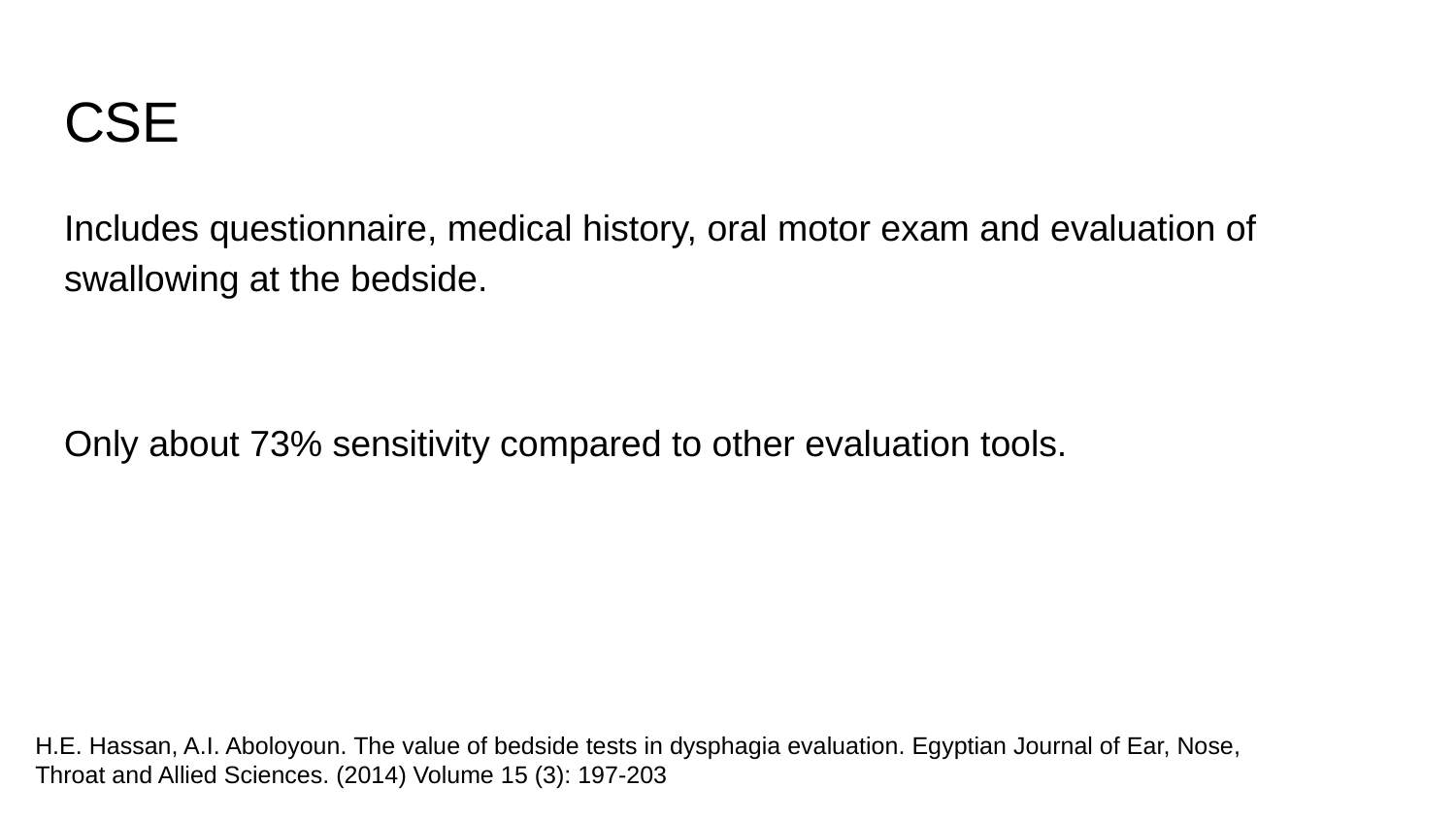

# CSE
Includes questionnaire, medical history, oral motor exam and evaluation of swallowing at the bedside.
Only about 73% sensitivity compared to other evaluation tools.
H.E. Hassan, A.I. Aboloyoun. The value of bedside tests in dysphagia evaluation. Egyptian Journal of Ear, Nose,
Throat and Allied Sciences. (2014) Volume 15 (3): 197-203

## Slide 10
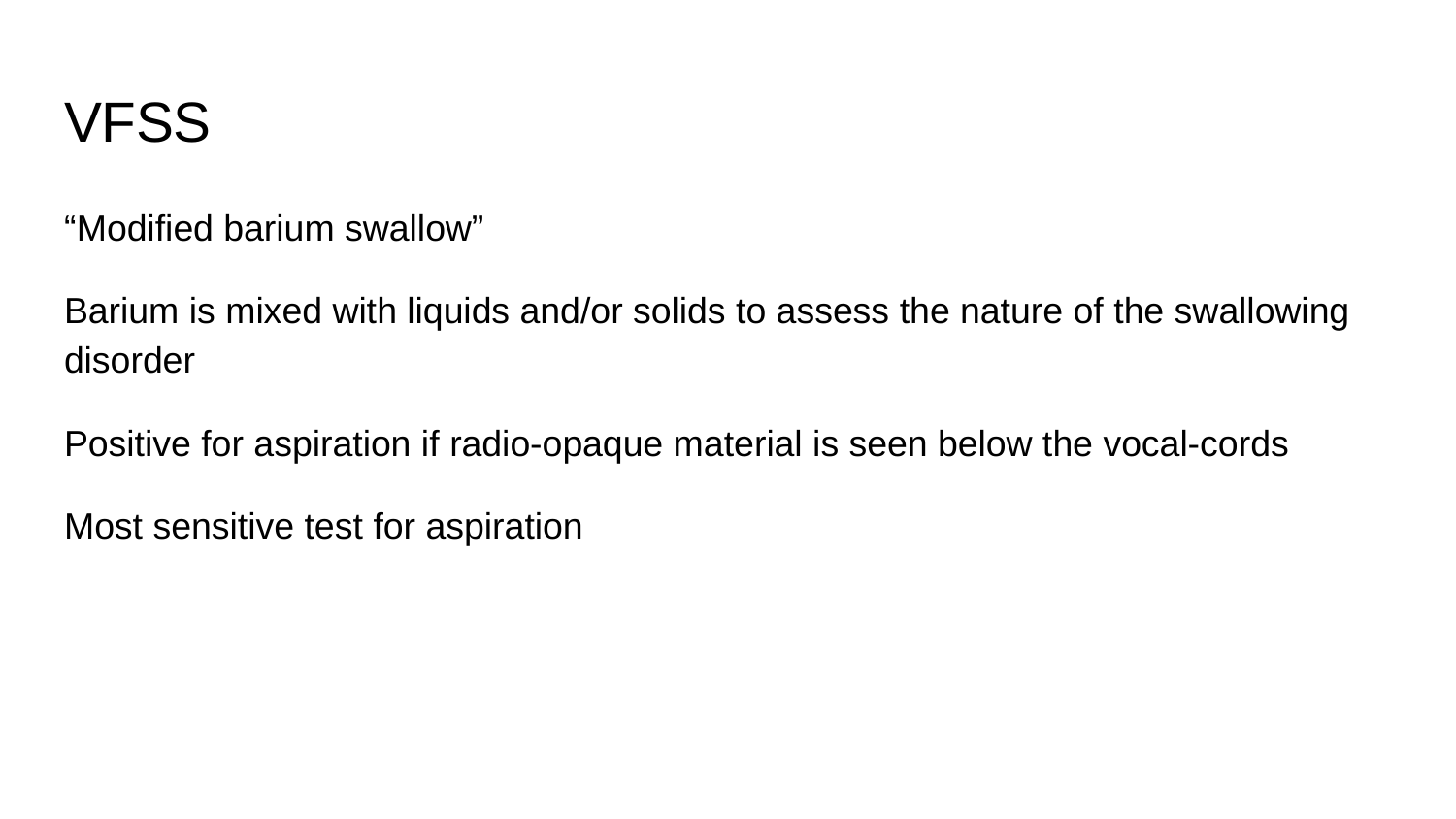

# VFSS
“Modified barium swallow”
Barium is mixed with liquids and/or solids to assess the nature of the swallowing disorder
Positive for aspiration if radio-opaque material is seen below the vocal-cords
Most sensitive test for aspiration

## Slide 11
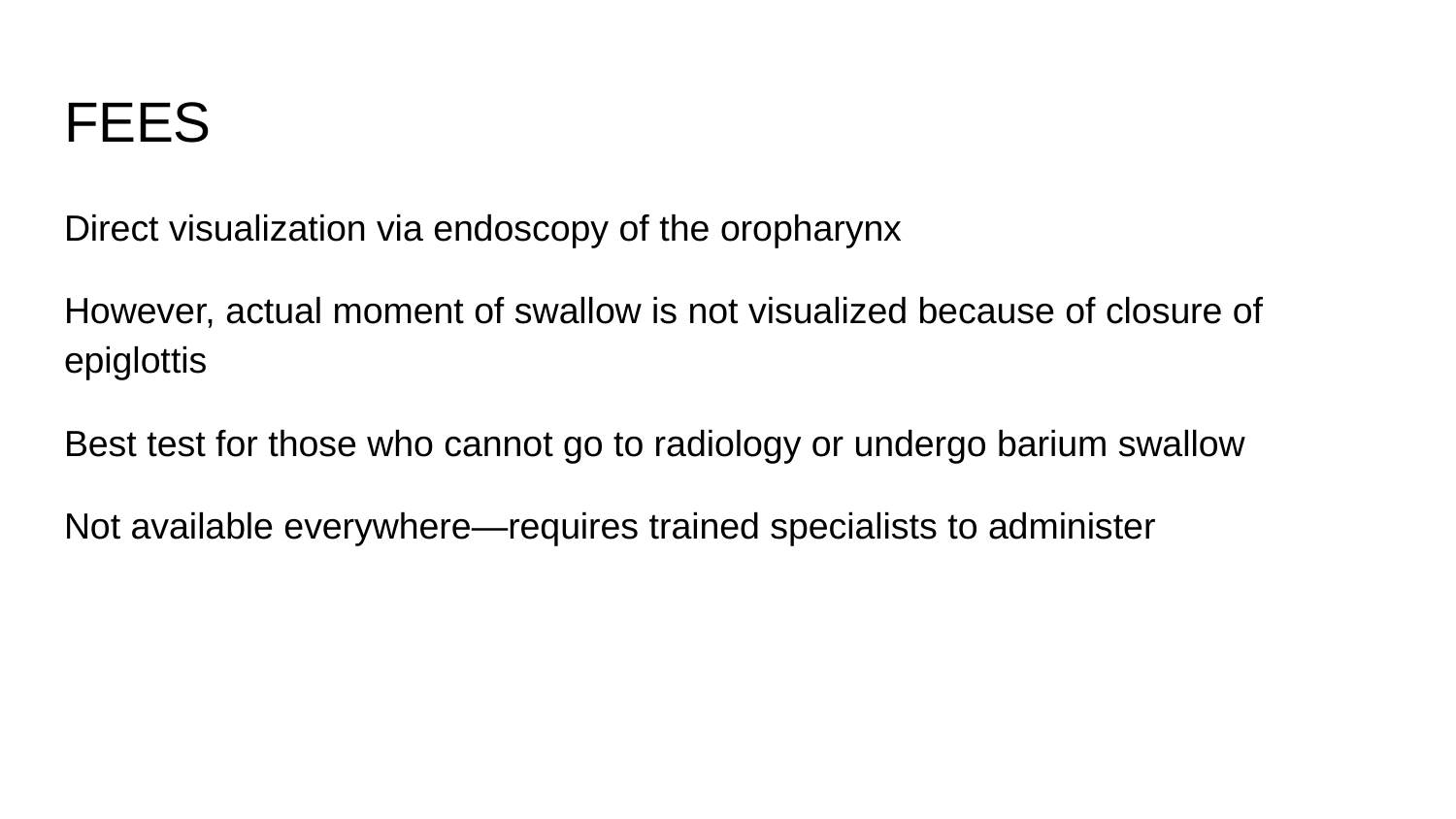

# FEES
Direct visualization via endoscopy of the oropharynx
However, actual moment of swallow is not visualized because of closure of epiglottis
Best test for those who cannot go to radiology or undergo barium swallow
Not available everywhere—requires trained specialists to administer

## Slide 12
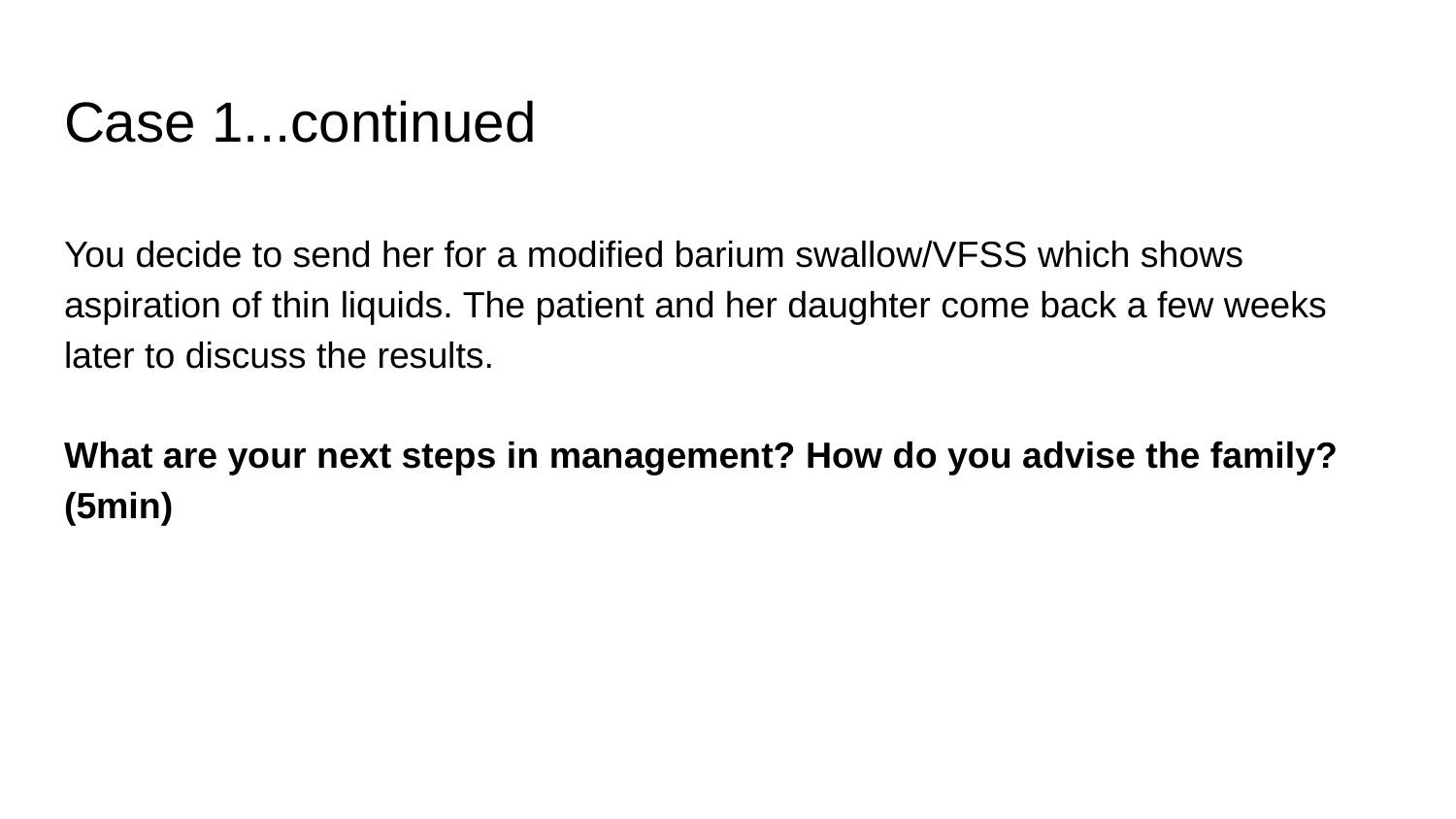

# Case 1...continued
You decide to send her for a modified barium swallow/VFSS which shows aspiration of thin liquids. The patient and her daughter come back a few weeks later to discuss the results.
What are your next steps in management? How do you advise the family? (5min)

## Slide 13
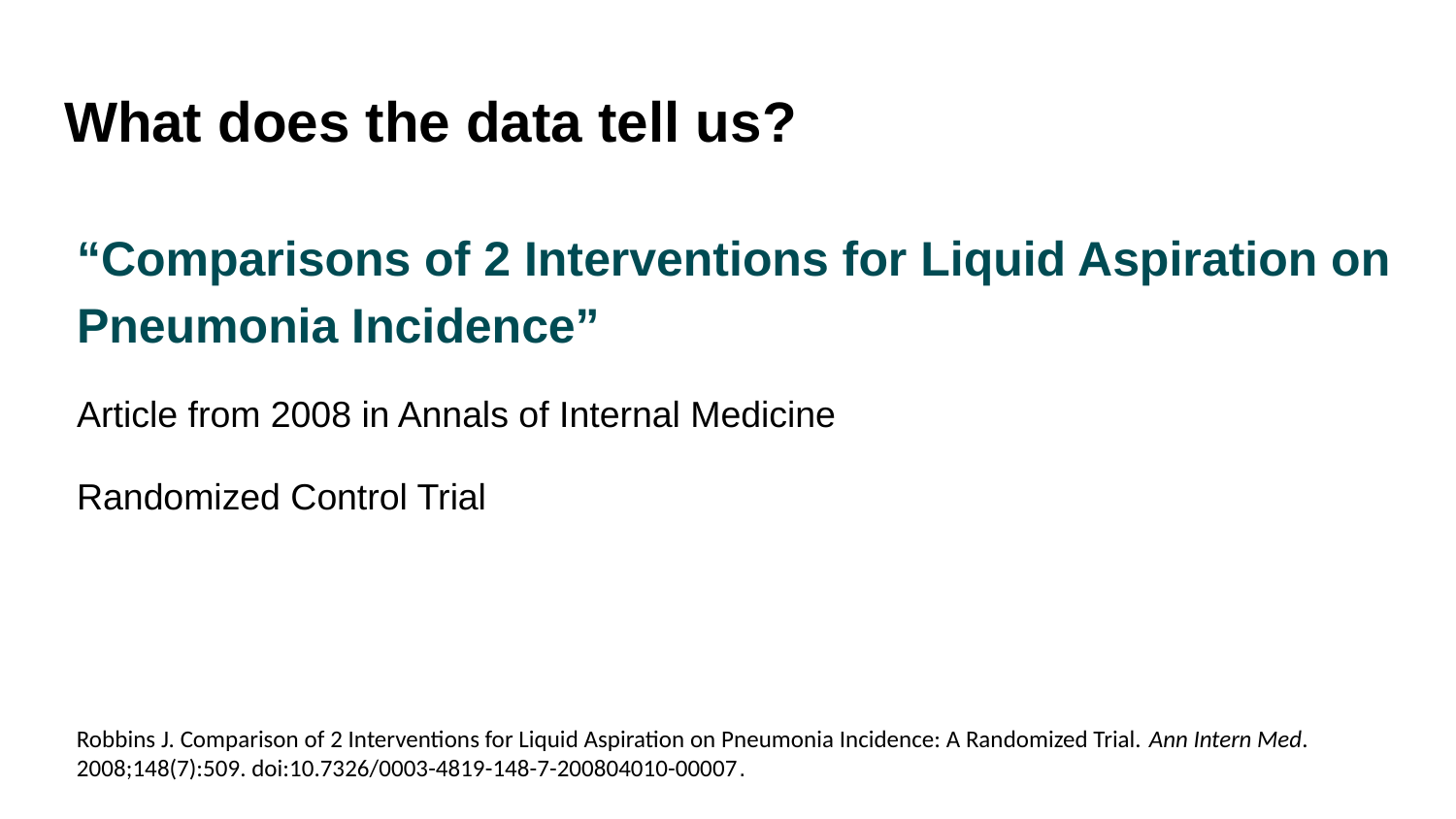

# What does the data tell us?
“Comparisons of 2 Interventions for Liquid Aspiration on Pneumonia Incidence”
Article from 2008 in Annals of Internal Medicine
Randomized Control Trial
Robbins J. Comparison of 2 Interventions for Liquid Aspiration on Pneumonia Incidence: A Randomized Trial. Ann Intern Med. 2008;148(7):509. doi:10.7326/0003-4819-148-7-200804010-00007.

## Slide 14
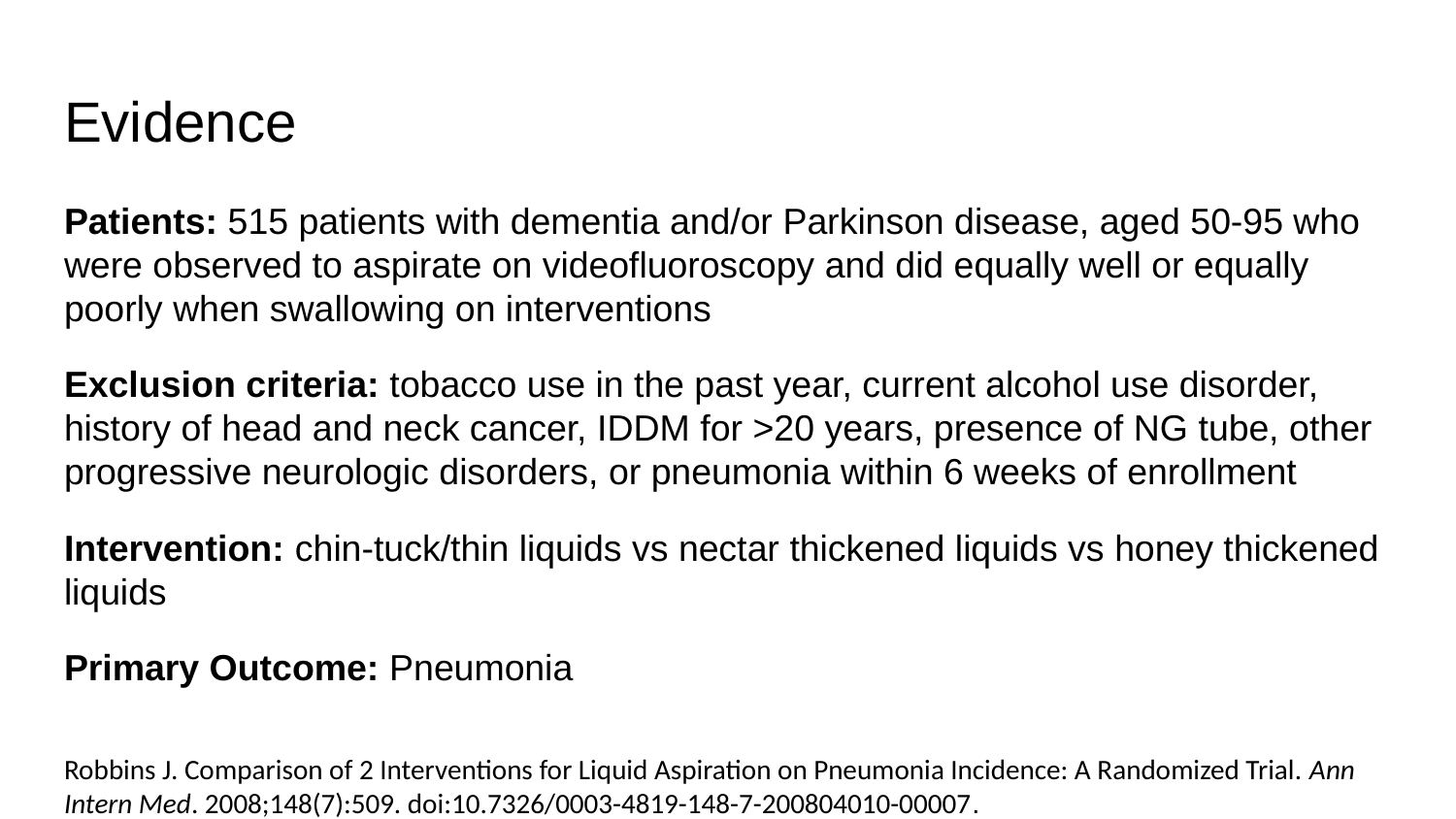

# Evidence
Patients: 515 patients with dementia and/or Parkinson disease, aged 50-95 who were observed to aspirate on videofluoroscopy and did equally well or equally poorly when swallowing on interventions
Exclusion criteria: tobacco use in the past year, current alcohol use disorder, history of head and neck cancer, IDDM for >20 years, presence of NG tube, other progressive neurologic disorders, or pneumonia within 6 weeks of enrollment
Intervention: chin-tuck/thin liquids vs nectar thickened liquids vs honey thickened liquids
Primary Outcome: Pneumonia
Robbins J. Comparison of 2 Interventions for Liquid Aspiration on Pneumonia Incidence: A Randomized Trial. Ann Intern Med. 2008;148(7):509. doi:10.7326/0003-4819-148-7-200804010-00007.

## Slide 15
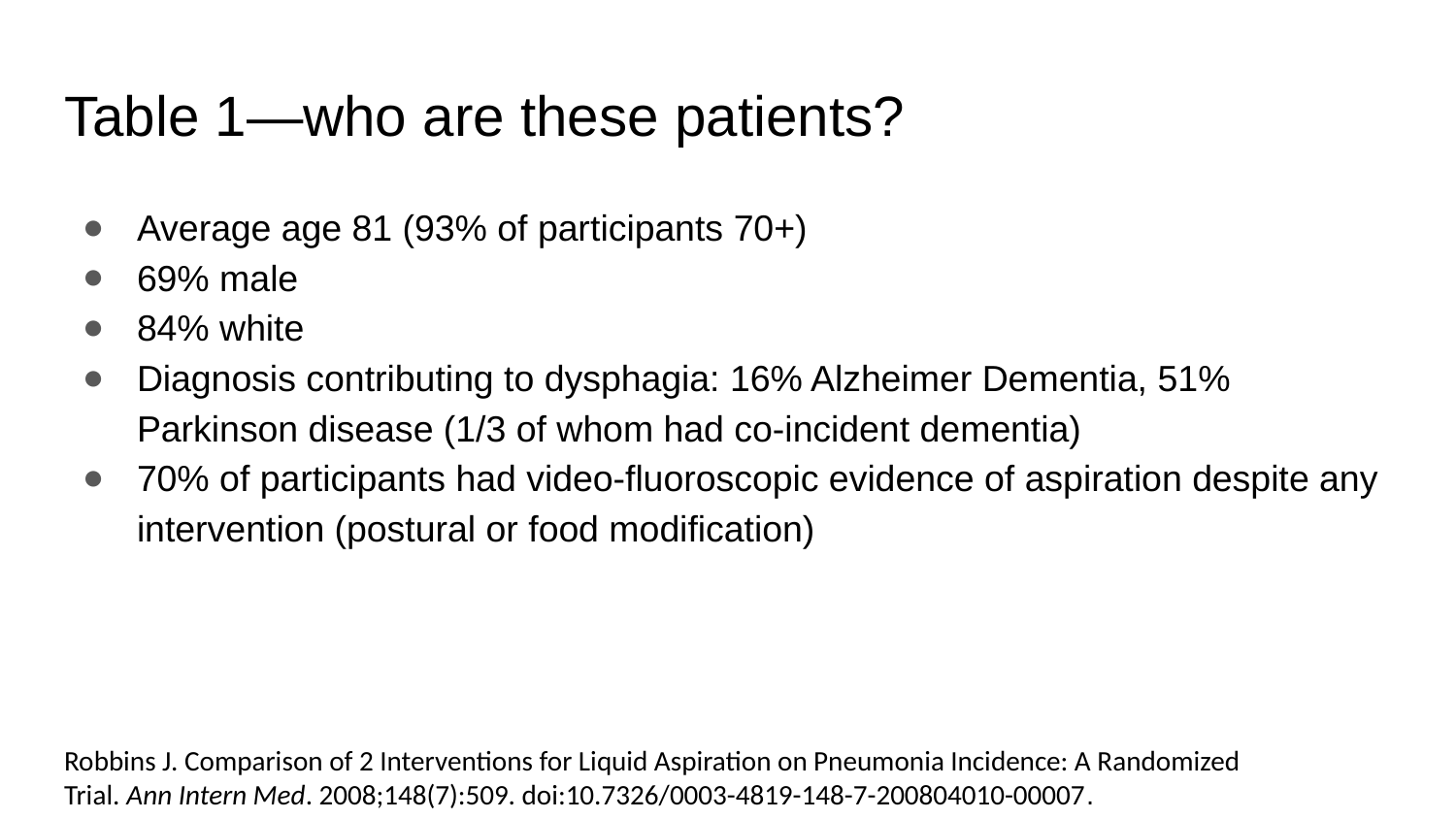

# Table 1—who are these patients?
Average age 81 (93% of participants 70+)
69% male
84% white
Diagnosis contributing to dysphagia: 16% Alzheimer Dementia, 51% Parkinson disease (1/3 of whom had co-incident dementia)
70% of participants had video-fluoroscopic evidence of aspiration despite any intervention (postural or food modification)
Robbins J. Comparison of 2 Interventions for Liquid Aspiration on Pneumonia Incidence: A Randomized Trial. Ann Intern Med. 2008;148(7):509. doi:10.7326/0003-4819-148-7-200804010-00007.

## Slide 16
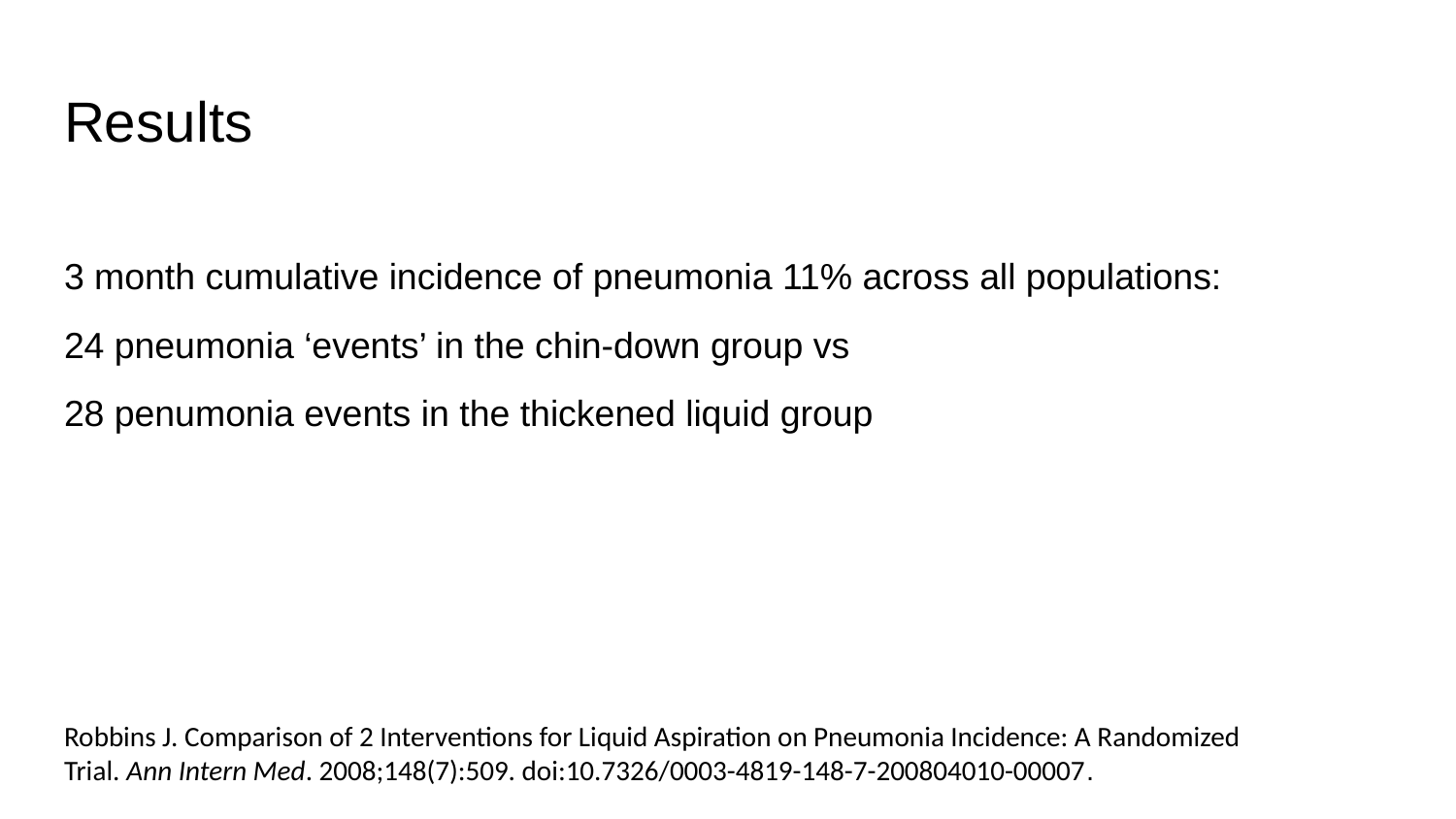

# Results
3 month cumulative incidence of pneumonia 11% across all populations:
24 pneumonia ‘events’ in the chin-down group vs
28 penumonia events in the thickened liquid group
Robbins J. Comparison of 2 Interventions for Liquid Aspiration on Pneumonia Incidence: A Randomized Trial. Ann Intern Med. 2008;148(7):509. doi:10.7326/0003-4819-148-7-200804010-00007.

## Slide 17
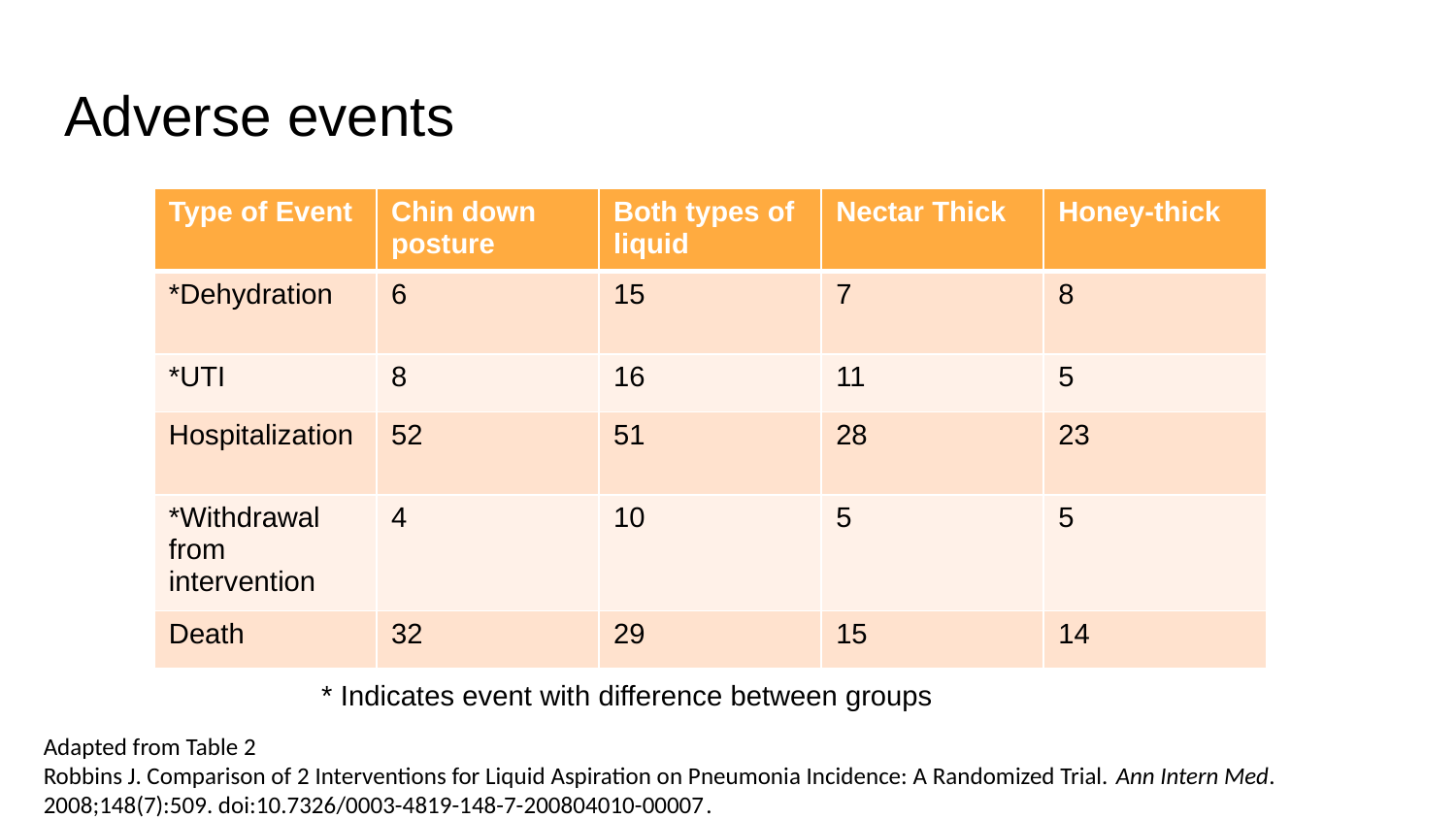

# Adverse events
| Type of Event | Chin down posture | Both types of liquid | Nectar Thick | Honey-thick |
| --- | --- | --- | --- | --- |
| \*Dehydration | 6 | 15 | 7 | 8 |
| \*UTI | 8 | 16 | 11 | 5 |
| Hospitalization | 52 | 51 | 28 | 23 |
| \*Withdrawal from intervention | 4 | 10 | 5 | 5 |
| Death | 32 | 29 | 15 | 14 |
* Indicates event with difference between groups
Adapted from Table 2
Robbins J. Comparison of 2 Interventions for Liquid Aspiration on Pneumonia Incidence: A Randomized Trial. Ann Intern Med. 2008;148(7):509. doi:10.7326/0003-4819-148-7-200804010-00007.

## Slide 18
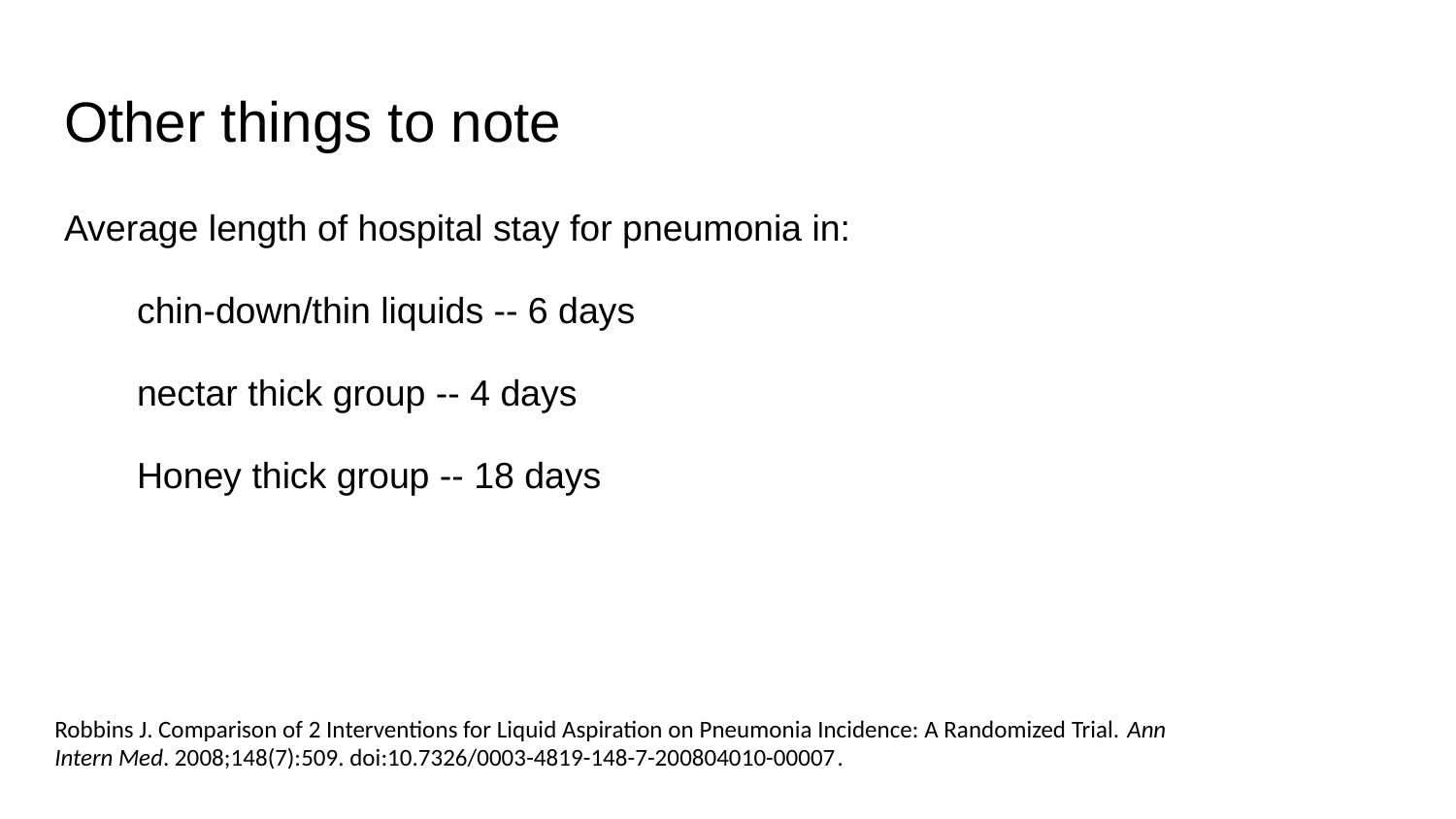

# Other things to note
Average length of hospital stay for pneumonia in:
chin-down/thin liquids -- 6 days
nectar thick group -- 4 days
Honey thick group -- 18 days
Robbins J. Comparison of 2 Interventions for Liquid Aspiration on Pneumonia Incidence: A Randomized Trial. Ann Intern Med. 2008;148(7):509. doi:10.7326/0003-4819-148-7-200804010-00007.

## Slide 19
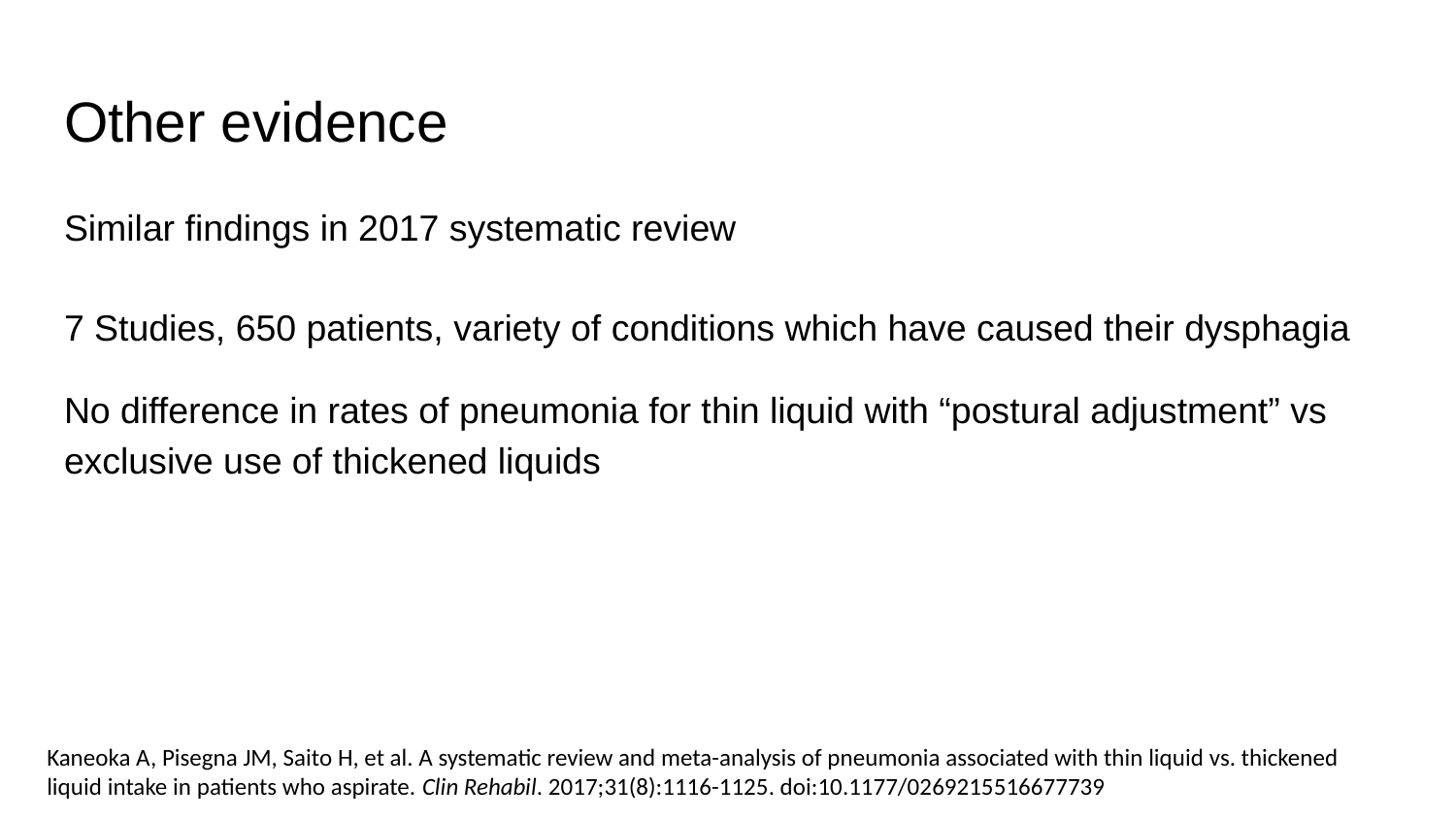

# Other evidence
Similar findings in 2017 systematic review
7 Studies, 650 patients, variety of conditions which have caused their dysphagia
No difference in rates of pneumonia for thin liquid with “postural adjustment” vs exclusive use of thickened liquids
Kaneoka A, Pisegna JM, Saito H, et al. A systematic review and meta-analysis of pneumonia associated with thin liquid vs. thickened liquid intake in patients who aspirate. Clin Rehabil. 2017;31(8):1116-1125. doi:10.1177/0269215516677739

## Slide 20
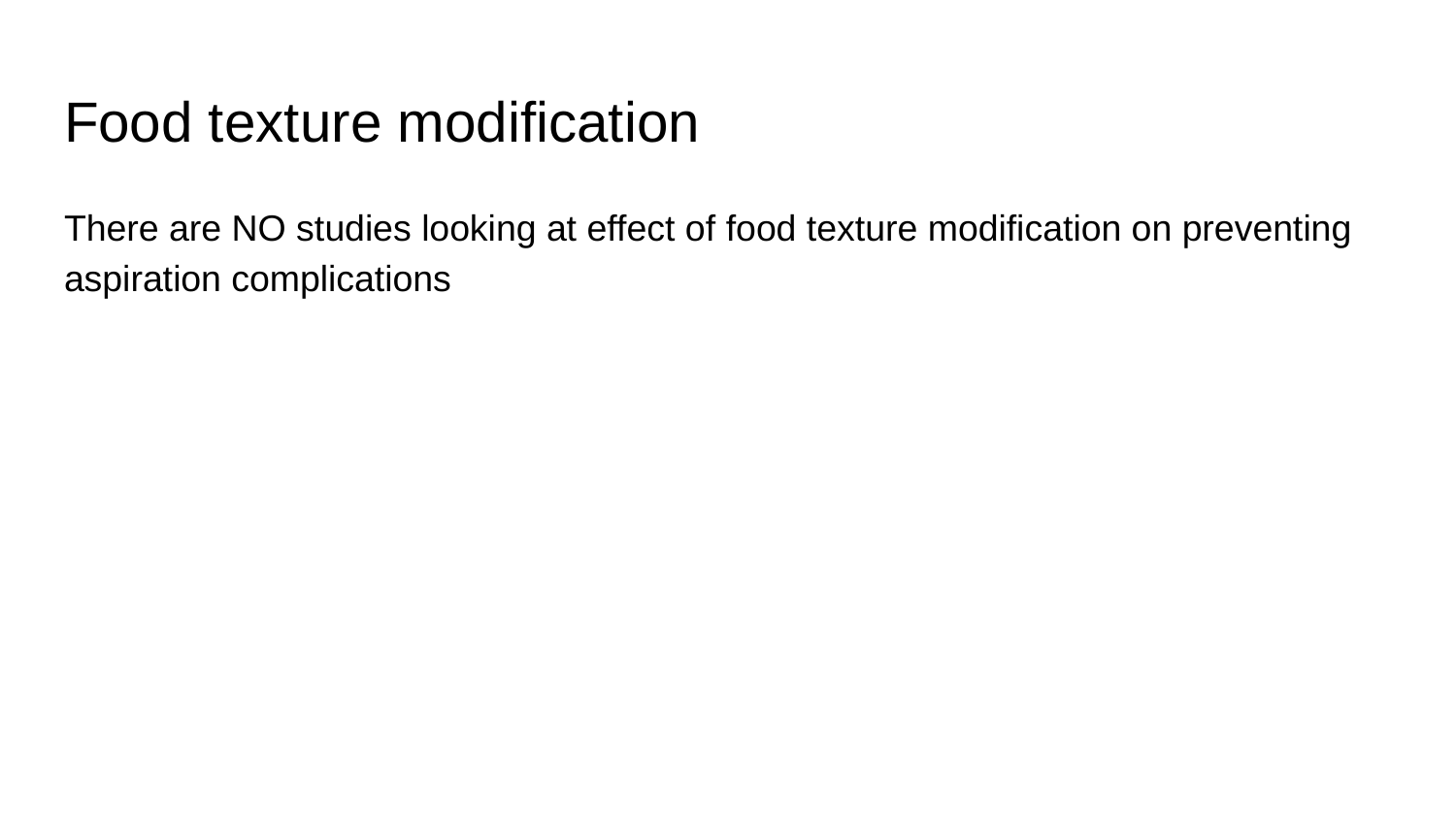

# Food texture modification
There are NO studies looking at effect of food texture modification on preventing aspiration complications

## Slide 21
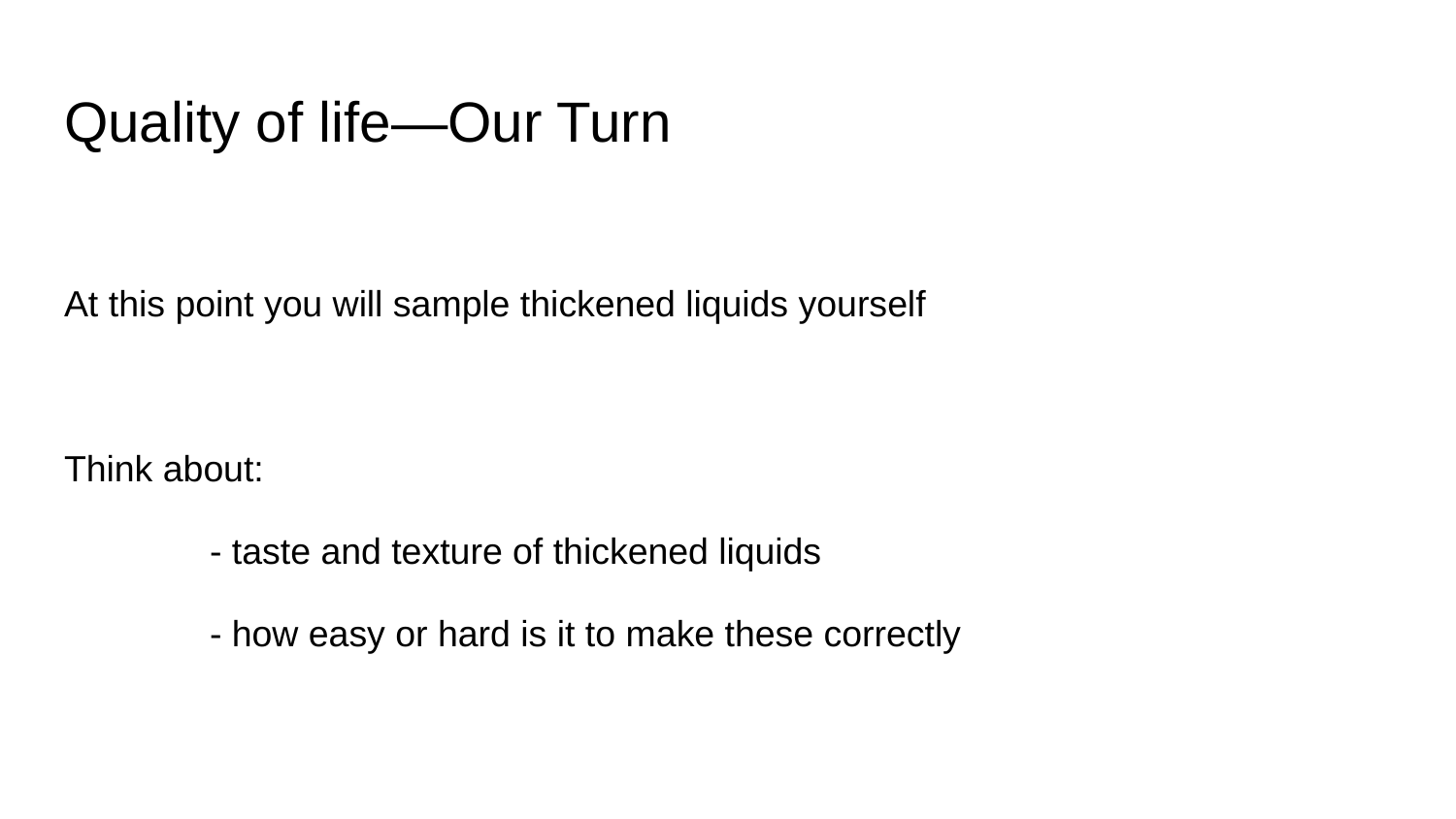

# Quality of life—Our Turn
At this point you will sample thickened liquids yourself
Think about:
	- taste and texture of thickened liquids
	- how easy or hard is it to make these correctly

## Slide 22
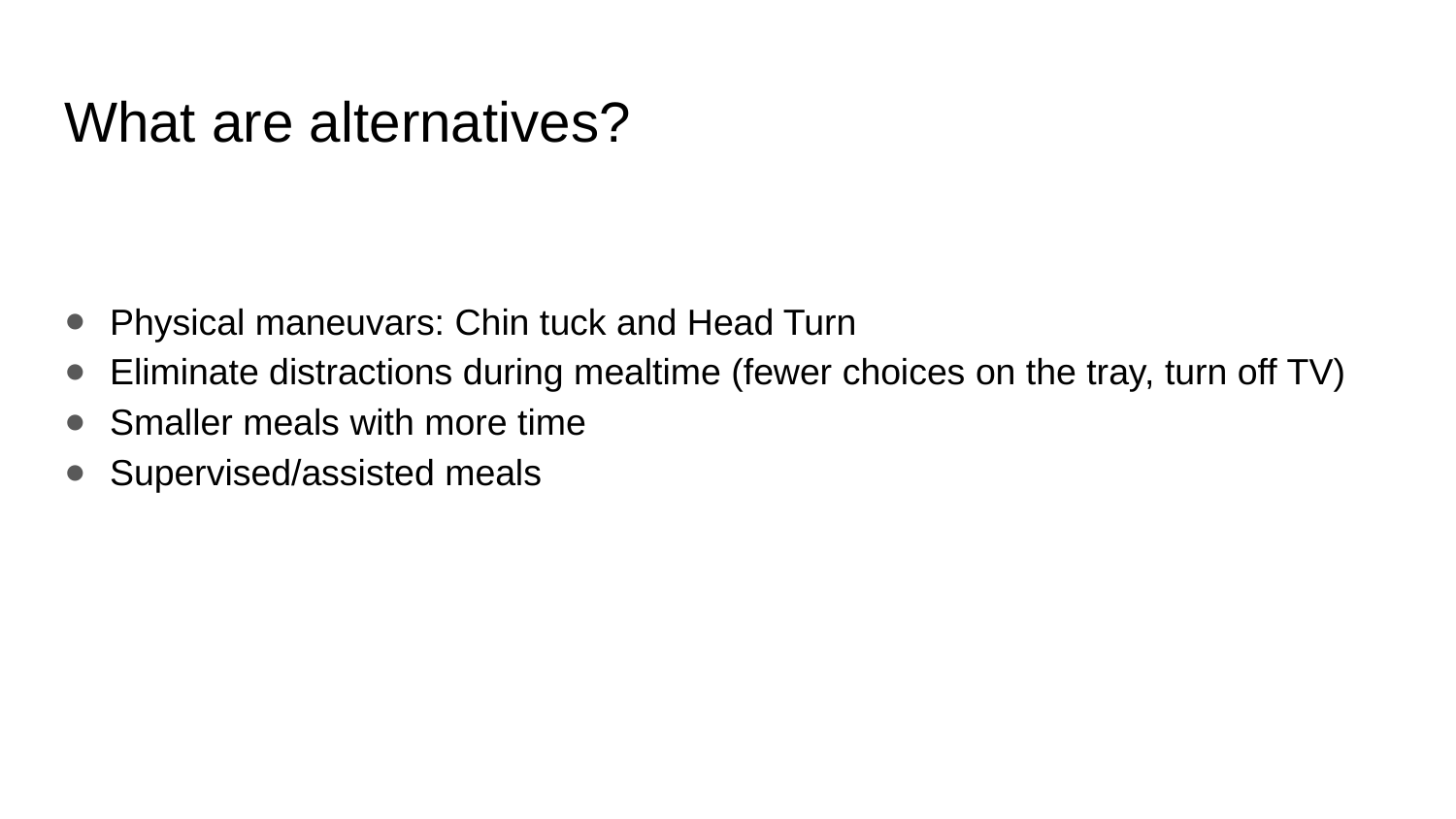

# What are alternatives?
Physical maneuvars: Chin tuck and Head Turn
Eliminate distractions during mealtime (fewer choices on the tray, turn off TV)
Smaller meals with more time
Supervised/assisted meals

## Slide 23
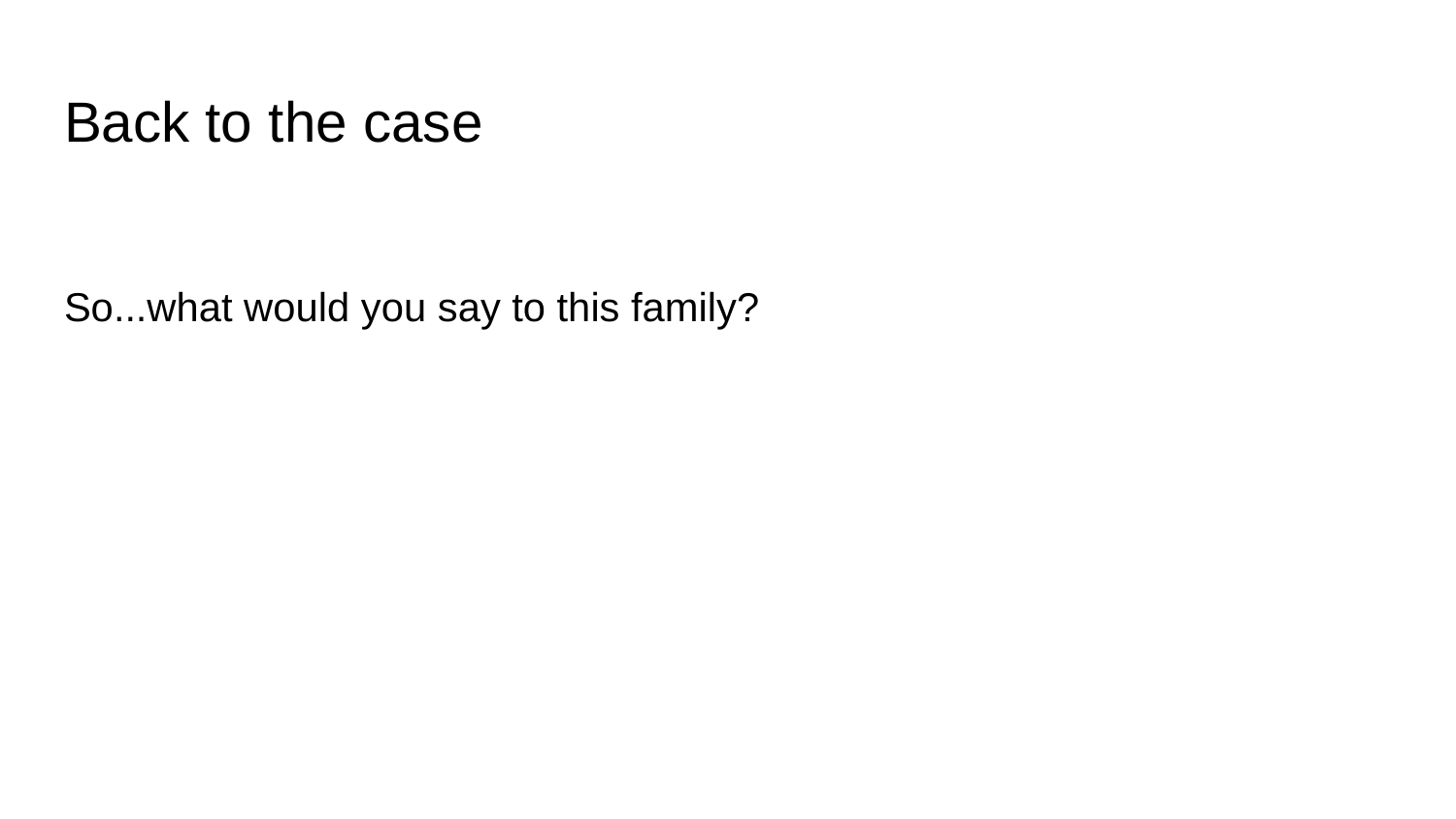

# Back to the case
So...what would you say to this family?

## Slide 24
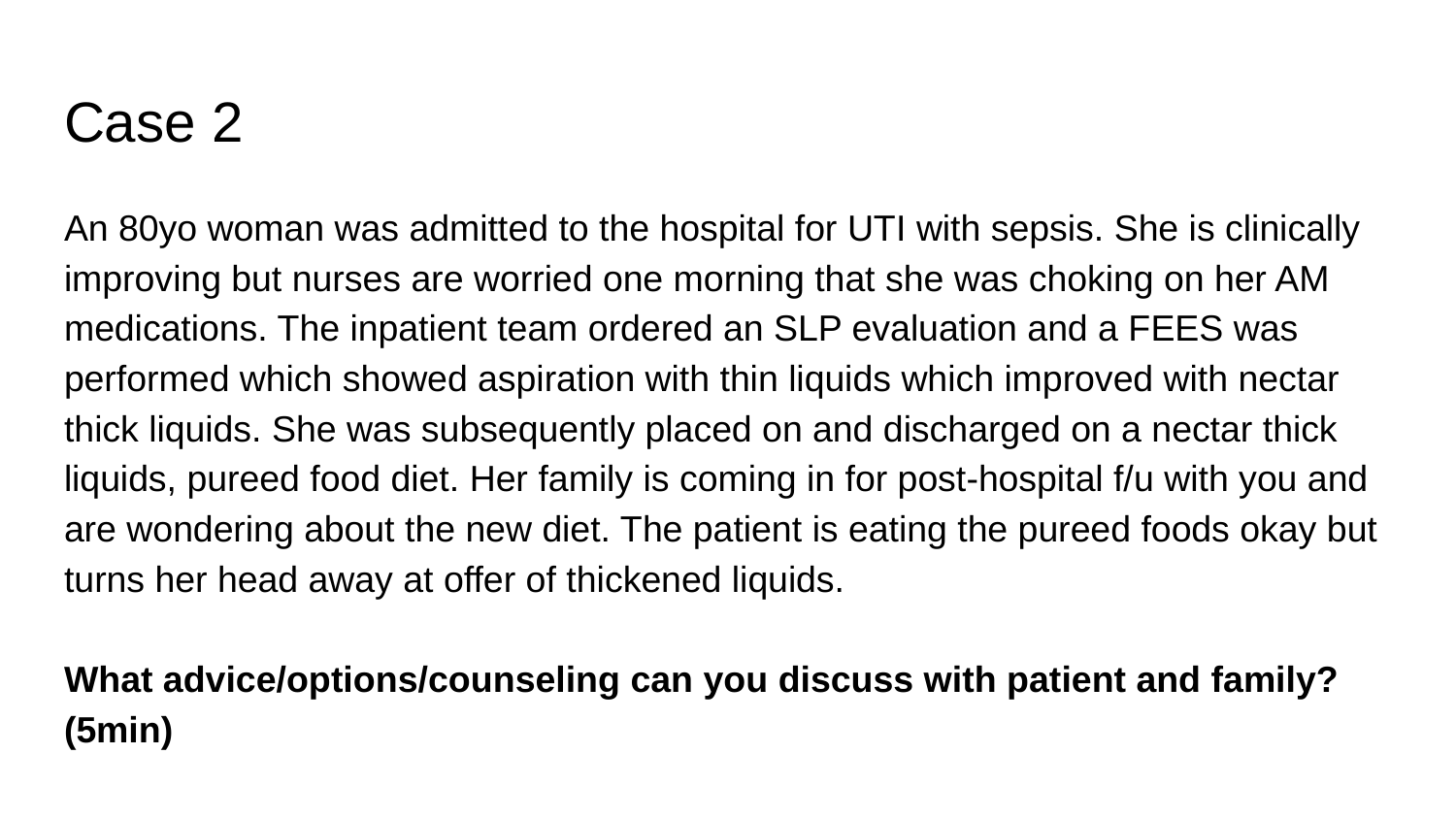

# Case 2
An 80yo woman was admitted to the hospital for UTI with sepsis. She is clinically improving but nurses are worried one morning that she was choking on her AM medications. The inpatient team ordered an SLP evaluation and a FEES was performed which showed aspiration with thin liquids which improved with nectar thick liquids. She was subsequently placed on and discharged on a nectar thick liquids, pureed food diet. Her family is coming in for post-hospital f/u with you and are wondering about the new diet. The patient is eating the pureed foods okay but turns her head away at offer of thickened liquids.
What advice/options/counseling can you discuss with patient and family? (5min)

## Slide 25
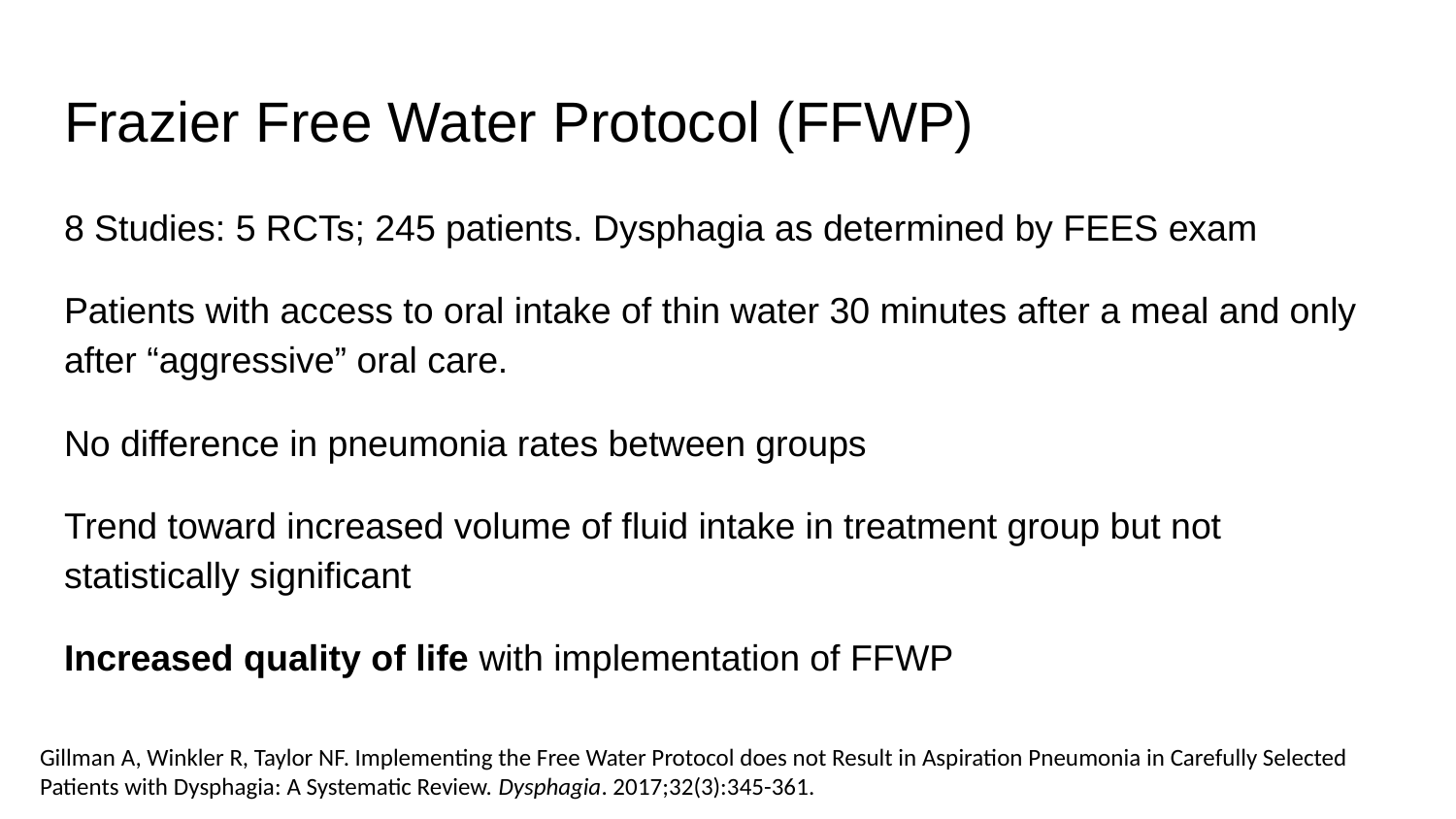

# Frazier Free Water Protocol (FFWP)
8 Studies: 5 RCTs; 245 patients. Dysphagia as determined by FEES exam
Patients with access to oral intake of thin water 30 minutes after a meal and only after “aggressive” oral care.
No difference in pneumonia rates between groups
Trend toward increased volume of fluid intake in treatment group but not statistically significant
Increased quality of life with implementation of FFWP
Gillman A, Winkler R, Taylor NF. Implementing the Free Water Protocol does not Result in Aspiration Pneumonia in Carefully Selected Patients with Dysphagia: A Systematic Review. Dysphagia. 2017;32(3):345-361.

## Slide 26
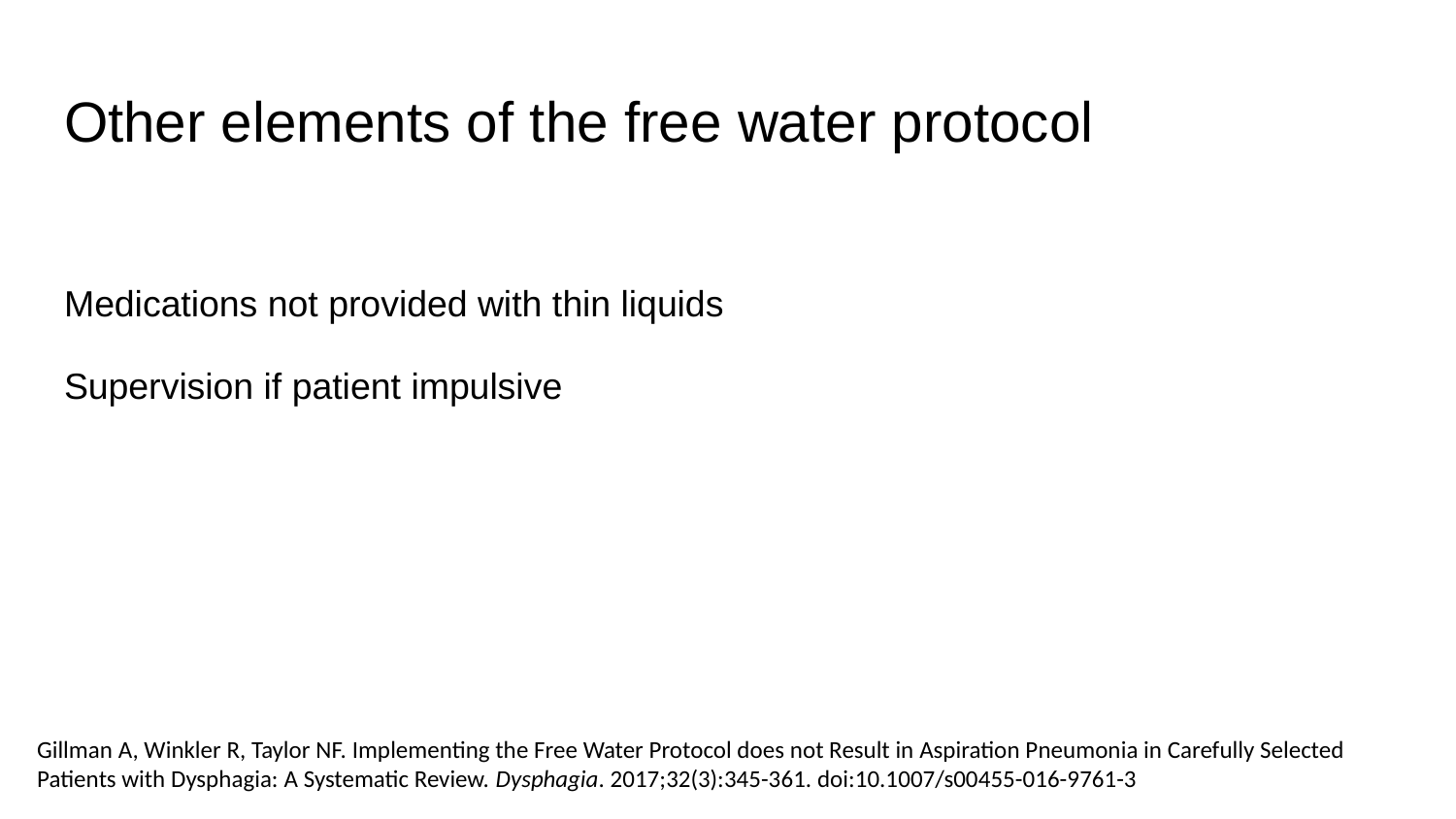

# Other elements of the free water protocol
Medications not provided with thin liquids
Supervision if patient impulsive
Gillman A, Winkler R, Taylor NF. Implementing the Free Water Protocol does not Result in Aspiration Pneumonia in Carefully Selected Patients with Dysphagia: A Systematic Review. Dysphagia. 2017;32(3):345-361. doi:10.1007/s00455-016-9761-3

## Slide 27
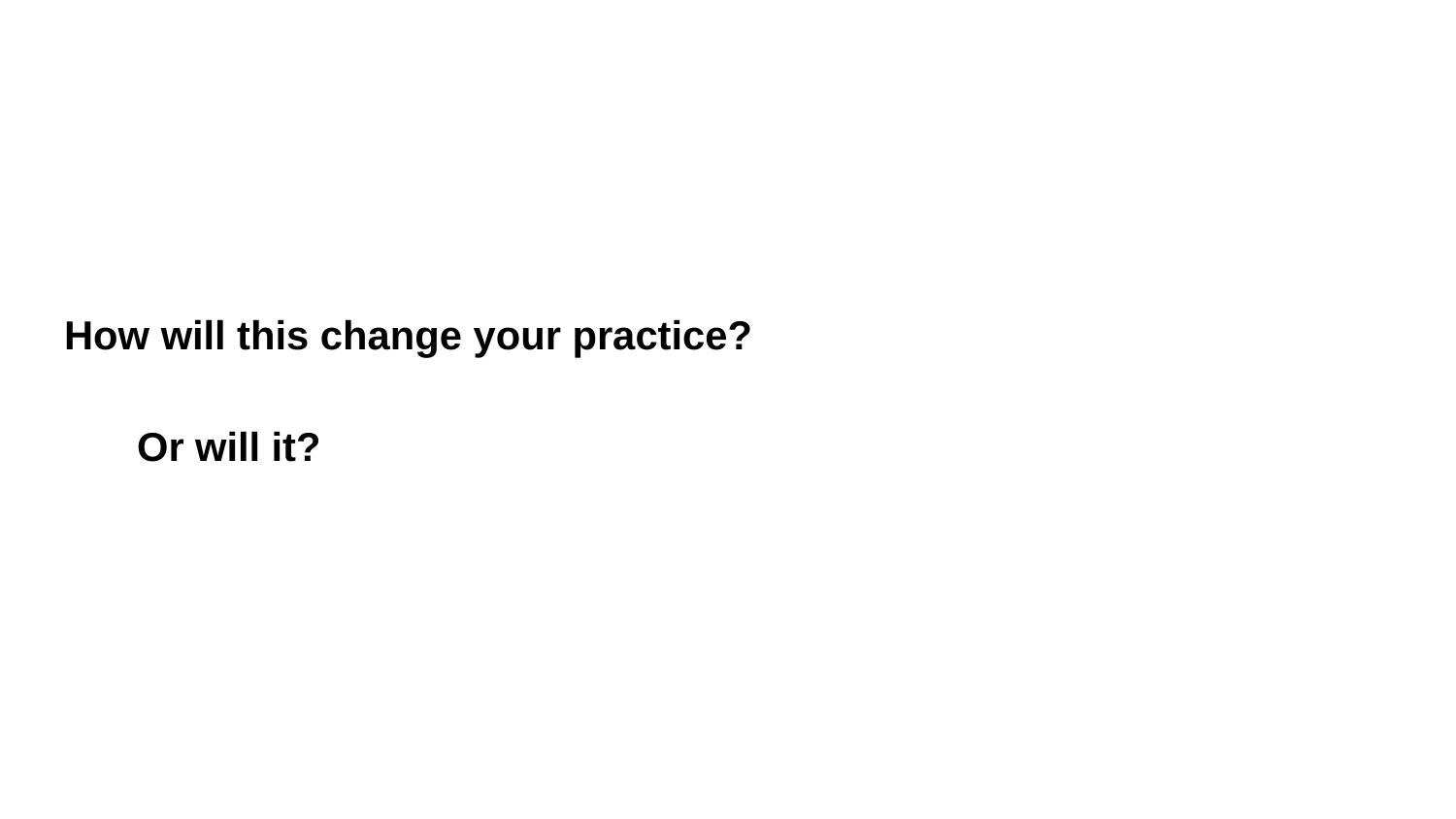

#
How will this change your practice?
Or will it?

## Slide 28
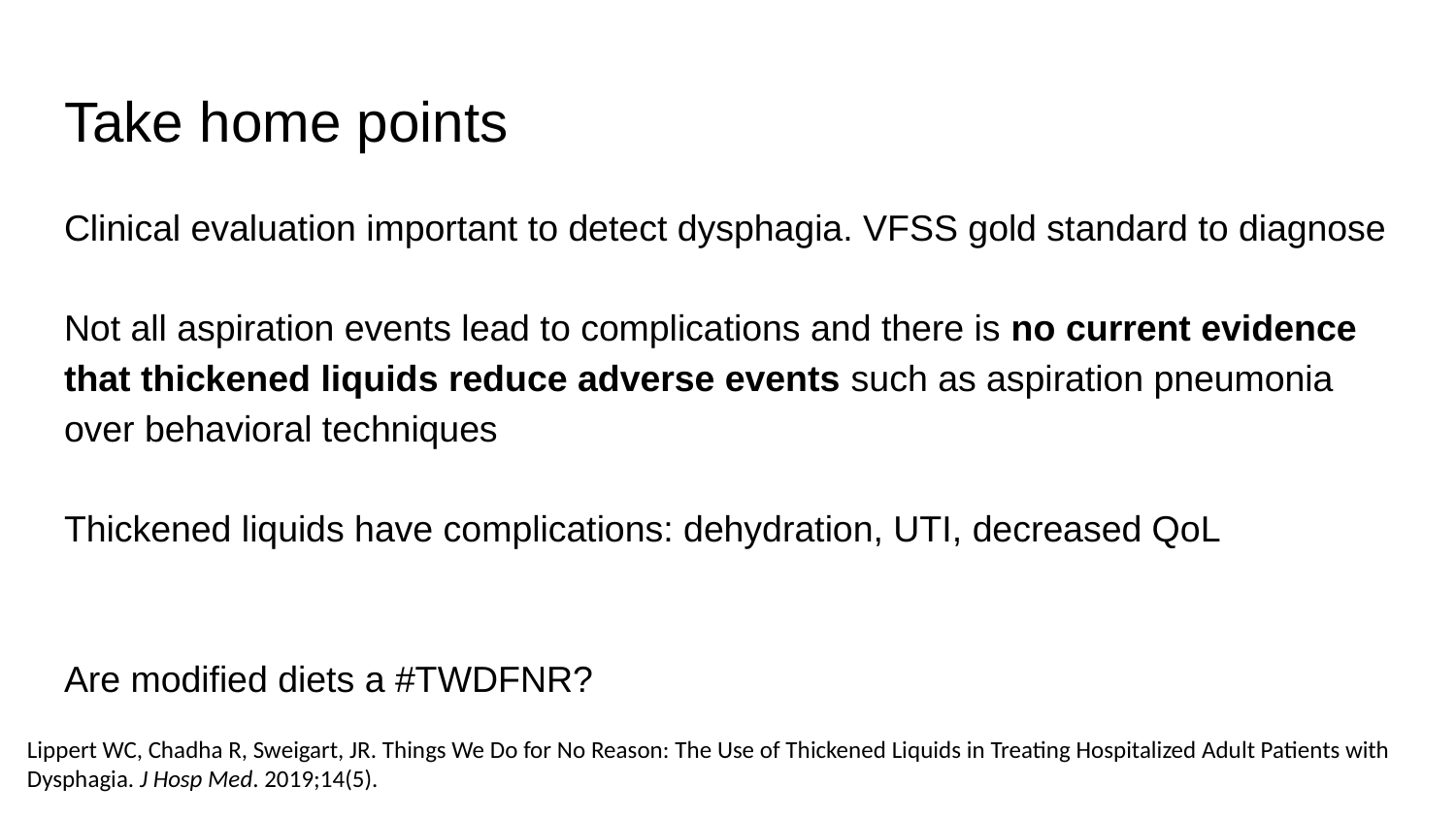

# Take home points
Clinical evaluation important to detect dysphagia. VFSS gold standard to diagnose
Not all aspiration events lead to complications and there is no current evidence that thickened liquids reduce adverse events such as aspiration pneumonia over behavioral techniques
Thickened liquids have complications: dehydration, UTI, decreased QoL
Are modified diets a #TWDFNR?
Lippert WC, Chadha R, Sweigart, JR. Things We Do for No Reason: The Use of Thickened Liquids in Treating Hospitalized Adult Patients with Dysphagia. J Hosp Med. 2019;14(5).
