# Supplementary material for: An Interactive Workshop on Managing Dysphagia in Older Adults With Dementia
Source: MedEdPORTAL. 2022 Mar 2;18:11223. doi: 10.15766/mep_2374-8265.11223 (PMC8888763; doi:10.15766/mep_2374-8265.11223)
Supplement: Supplementary file 1 — Pre- and Postsurvey.docxDysphagia in Dementia.pptxFacilitator Guide.docx [file mep_2374-8265.11223-s001.zip › A. Pre- and Postsurvey.docx]

**Background Information:**

Please circle your PGY Level:

PGY1 PGY2 PGY3

What is your most likely future career path? *Please select one*

- Primary care/General Internal Medicine
- Primary care Subspecialty (eg geriatrics, addiction med)
- IM subspecialty (eg rheum, endo, cards, GI)
- GIM Hospitalist
- Undetermined

**Prior Experience:**

Have you had any formal teaching to date (e.g., Lecture, attending rounds, workshops) on the management of patients with dysphagia (e.g., diagnostics, modified diets, treatment)?

Yes No

If yes, please briefly describe the educational experience (e.g., setting-medical school, residency, other and content – lecture by SLP, etc.):

__________________________________________________________________________________________________________________________________________________________________________

**Current Experience:**

How often do you encounter patients with dysphagia in your current practice (inpatient OR outpatient): *Please select one.*

| Never | Rarely (less than once every 6 months) | Once every 3-6 months | Once every 1-3 months | More than once a month |
| --- | --- | --- | --- | --- |

How comfortable do you feel managing patients with dementia with dysphagia: *Please select one.*

| I do not feel comfortable managing patients with dysphagia | I feel comfortable managing patients with dysphagia with A LOT of assistance from a speech and language pathologist (SLP) or preceptor | I feel comfortable managing dysphagia with SOME guidance and prompting by a SLP or preceptor | I feel comfortable managing dysphagia with LITTLE assistance from a SLP or preceptor | I feel comfortable managing patients with dysphagia independently |
| --- | --- | --- | --- | --- |

It is important for me to be able to manage patients with dysphagia in my **CURRENT** practice as an internal medicine resident.

| Not important at all | Less important | Unsure | Somewhat important | Very important |
| --- | --- | --- | --- | --- |

It is important for me to able to manage patients with dysphagia in my **FUTURE** practice.

| Not important at all | Less important | Unsure | Somewhat important | Very important |
| --- | --- | --- | --- | --- |

What is your general approach to a patient with dementia who is experiencing difficulty swallowing in the inpatient setting:

What is your general approach to a patient with dementia who is experiencing difficulty swallowing in the outpatient setting:

**Knowledge Check-in: [Facilitator Note: Correct answers marked with *]**

1. In what stage of Alzheimer’s dementia does dysphagia typically present?

Mild Moderate * Advanced Variable

1. The ability to organize sequenced movements of eating correlates with the severity of dementia.

True* False

1. What percentage of aspiration events are silent (not evident by clinical exam)? *Please select one.*

10% 30% * 50% 80%

1. What is the most sensitive diagnostic evaluation for dysphagia? *Please select one.*

bedside swallow video fluoroscopy (“modified barium swallow”)* FEES exam

5. Patients on a modified diet are significantly more likely to: *Select all that apply*

-be prescribed supplemental nutrition *

-take in fewer calories *

-develop pressure ulcers

-have better quality of life

1. Evidence of dysphagia on video-fluoroscopic exam correlates with risk of aspiration pneumonia.

True False *

1. Rates of aspiration pneumonia are higher in those who utilize a chin tuck maneuver and drink thin liquids vs those who do not utilize chin tuck maneuver and drink thickened liquids**.**

True False *

Please list any evidence-based behavioral alternatives to thickened liquids that you are aware of to help prevent aspiration in patients with dementia and dysphagia.

__________________________________________________________________________________________________________________________________________________________________________

8. Which of these is/are adverse effects of a texture modified/ thickened diet? (check all that apply)

-dehydration *

-UTI *

-lower scores on Quality of Life metrics *

-diarrhea

9. For those otherwise on a texture modified diet, access to free water between meals correlated with: (check all that apply)

-lower rates of dehydration*

-higher rates of pneumonia

-higher QoL scores *

-no difference in QoL scores

**Please rate the extent to which you agree or disagree with each of the following statements.**

|  | **Strongly**  **Agree** | **Agree** | **Somewhat Agree** | **Neither Agree nor Disagree** | **Somewhat Disagree** | **Disagree** | **Strongly Disagree** |
| --- | --- | --- | --- | --- | --- | --- | --- |
| **Thickened liquids help prevent aspiration events for people with dysphagia.** |  |  |  |  |  |  |  |
| **Thickened liquids help prevent pneumonia for people with dysphagia** |  |  |  |  |  |  |  |
| **Thickened liquids can cause adverse events** |  |  |  |  |  |  |  |
| **Aspiration always leads to pneumonia** |  |  |  |  |  |  |  |
| **Patients don’t mind being on a modified diet** |  |  |  |  |  |  |  |
