# Supplementary material for: An Interactive Workshop on Managing Dysphagia in Older Adults With Dementia
Source: MedEdPORTAL. 2022 Mar 2;18:11223. doi: 10.15766/mep_2374-8265.11223 (PMC8888763; doi:10.15766/mep_2374-8265.11223)
Supplement: Supplementary file 1 — Pre- and Postsurvey.docxDysphagia in Dementia.pptxFacilitator Guide.docx [file mep_2374-8265.11223-s001.zip › C. Facilitator Guide.docx]

**Dysphagia in Patients with Dementia Workshop Facilitator’s Guide**

*Authors: Nicole Mushero, MD, PhD, Lindsay Demers, PhD, Ryan Chippendale, MD*

**Introduction**

There are more than 5 million adults living with dementia in the United States and that number is projected to increase exponentially over the next 30 years. Dysphagia is a common phenomenon which accompanies dementia and impacts both nutrition and quality of life. There is a shortfall in the number of geriatricians available to care for older adults living with dementia. Thus, primary care providers should be equipped with the knowledge to adequately care for this vulnerable population. We created this brief, interactive, case- based session to educate internal medicine resident physicians on how to evaluate and treat dementia associated dysphagia.

This workshop consists of a case-based approach mixed with small-group breakouts and mini-didactics, in addition to a hands-on experience with testing different consistencies of modified liquid diets. A pre-post survey design can be used to assess learners’ knowledge, attitudes and confidence toward managing patients that have dysphagia and dementia.

**Goals and Objectives**

By the end of this session, participating trainees will be able to:

1. Appropriately evaluate patients with dementia for dysphagia
2. Describe the available evidence regarding dietary modifications for dysphagia
3. Evaluate the risks associated with thickened liquids
4. Facilitate conversations with patients and families around the risks and benefits of dietary modifications and alternatives

*Participants:* Internal medicine residents or other primary-care based specialty trainees (e.g., medical students, nurse practitioners, physician assistants) or trainees that are likely to encounter patients with dementia and dysphagia (e.g., Neurology)

*Faculty:* At least one Geriatrician or other clinician well-versed in dementia and dysphagia

*Session Structure:*

- One-time session of 75 minutes duration
- Room overview: Participants are in groups around tables (4-5 participants per group) from which they can also view the slide show and facilitator/presenter. The groupings at table allow for easy small group breakouts throughout the session.

*Materials:*

- Pre-survey & consent form for participants (electronic)
- Post-survey for participants (electronic)
- Thickened liquid powder (can be purchased at any pharmacy or online) and juice, cups, spoons
- Projector, computer and PowerPoint presentation

*Faculty Preparation:*

Prior to the session, faculty should review the learning objectives, small group breakout cases, slide presentation and facilitator’s guide. It is also encouraged to review the article cited in slides 13-17 of the slide presentation.

*Session Flow:*

1. Min 1-5 (Slides 1-2): introduction of faculty member(s) and review of the purpose and learning objectives for the session.
2. Min 6-35 (Slides 3-20): introduction of first case, small group breakouts, large group debrief, and didactic teaching focused around case-based question
3. Min 36-50 (Slide 21): Residents’ sampling thickened liquids to understand challenges of use and impact on taste/quality. There should be enough cups and juice for all residents to sample. Learners should realize complexity of making appropriately thickened beverages based on the instructions on the canister and trial thickened liquids (water or juice) to understand impact on taste/texture.
4. Min 50-60 (Slide 22-23): Finish case 1, practicing actual phrases that learners could use in discussion with family (See Slide 23 speaker notes for examples)
5. Min 61-70 (Slides 24-26): Introduction of second case, time split between small groups and didactic teaching
6. Min 71-75 (Slides 27-28): Wrap up, review of key take-home points/learning objectives, final questions

If desired and time allows, 5-10 minutes at the beginning and end of the session for completion of the pre- and post-surveys can be added.
